# USP25 promotes pathological HIF-1-driven metabolic reprogramming and is a potential therapeutic target in pancreatic cancer

**DOI:** 10.1038/s41467-022-29684-9

**Published:** 2022-04-19

**Authors:** Jessica K. Nelson, May Zaw Thin, Theodore Evan, Steven Howell, Mary Wu, Bruna Almeida, Nathalie Legrave, Duco S. Koenis, Gabriela Koifman, Yoichiro Sugimoto, Miriam Llorian Sopena, James MacRae, Emma Nye, Michael Howell, Ambrosius P. Snijders, Andreas Prachalias, Yoh Zen, Debashis Sarker, Axel Behrens

**Affiliations:** 1 Adult Stem Cell Laboratory, The Francis Crick Institute, 1 Midland Road, London, NW1 1AT UK; 2 Cancer Stem Cell Laboratory, The Breast Cancer Now Toby Robins Research Centre, Institute of Cancer Research, 237 Fulham Road, London, SW3 6JB UK; 3 Proteomics, The Francis Crick Institute, 1 Midland Road, London, NW1 1AT UK; 4 High Throughput Screening, The Francis Crick Institute, 1 Midland Road, London, NW1 1AT UK; 5 Experimental Histopathology, The Francis Crick Institute, 1 Midland Road, London, NW1 1AT UK; 6 Metabolomics, The Francis Crick Institute, 1 Midland Road, London, NW1 1AT UK; 7 William Harvey Research Institute, Barts and The London School of Medicine and Dentistry, Queen Mary University of London, Charterhouse Square, London, EC1M 6BQ UK; 8 Hypoxia Biology Laboratory, The Francis Crick Institute, 1 Midland Road, London, NW1 1AT UK; 9 Bioinformatics and Biostatistics, The Francis Crick Institute, 1 Midland Road, London, NW1 1AT UK; 10 Hepatobiliary and Pancreatic Surgery, King’s College Hospital, Denmark Hill, London, SE5 9RS UK; 11 Institute of Liver Studies, King’s College Hospital, Denmark Hill, London, SE5 9RS UK; 12 School of Cancer and Pharmaceutical Sciences, King’s College Hospital, Denmark Hill, London, SE5 9RS UK; 13 Imperial College, Division of Cancer, Department of Surgery and Cancer, Imperial College, Exhibition Road, London, SW7 2AZ UK; 14 Convergence Science Centre, Imperial College, Exhibition Road, London, SW7 2BU UK

**Keywords:** Cancer metabolism, Cancer models, Drug development, Pancreatic cancer, Deubiquitylating enzymes

## Abstract

Deubiquitylating enzymes (DUBs) play an essential role in targeted protein degradation and represent an emerging therapeutic paradigm in cancer. However, their therapeutic potential in pancreatic ductal adenocarcinoma (PDAC) has not been explored. Here, we develop a DUB discovery pipeline, combining activity-based proteomics with a loss-of-function genetic screen in patient-derived PDAC organoids and murine genetic models. This approach identifies USP25 as a master regulator of PDAC growth and maintenance. Genetic and pharmacological USP25 inhibition results in potent growth impairment in PDAC organoids, while normal pancreatic organoids are insensitive, and causes dramatic regression of patient-derived xenografts. Mechanistically, USP25 deubiquitinates and stabilizes the HIF-1α transcription factor. PDAC is characterized by a severely hypoxic microenvironment, and *USP25* depletion abrogates HIF-1α transcriptional activity and impairs glycolysis, inducing PDAC cell death in the tumor hypoxic core. Thus, the USP25/HIF-1α axis is an essential mechanism of metabolic reprogramming and survival in PDAC, which can be therapeutically exploited.

## Abstract (web summary)

The biological roles of deubiquitinating enzymes (DUBs) in pancreatic ductal adenocarcinoma (PDAC) are not fully explored. Here the authors perform activity based proteomics with a loss of function genetic screen and identify that USP25 promotes PDAC growth and survival through HIF-1 protein stability and transcriptional activity.

## Introduction

Pancreatic ductal adenocarcinoma (PDAC) is a highly lethal disease with a 5-year survival rate below 9%^1^, and projected to be the second-leading cause of cancer death by 2030^2^. PDAC tumor aggressiveness and resistance to traditional chemotherapy are believed to arise from a lack of clinically targetable mutations, an immune cell-depleted hypoxic microenvironment, altered metabolism, and tumor heterogeneity. Malignant progression from pancreatic intra-epithelial neoplasia (PanINs) to invasive and metastatic disease is usually accompanied by early acquisition of activating mutations in the *KRAS* oncogene, which occurs in >90% of all PDAC cases, followed by loss of tumor suppressors such as *INK4A/ARF*, *TP53*, and *SMAD4*^3^. Despite this knowledge of the PDAC genetic signature, therapeutic efforts to inhibit the key oncogenic driver KRAS have been largely unsuccessful to date. Therefore, new therapeutic strategies that specifically target cancer-specific pathways are urgently needed for the successful treatment of this disease.

Recently, the emergence of three-dimensional tumor organoid models with the intrinsic advantage of retaining the heterogeneity of original tumors has allowed for improved modeling of PDAC^4–8^. Characterization efforts in organoid models have led to a more refined understanding of the mechanisms involved in PDAC development^5^, as well as better correlation with therapeutic responses in patients^6,7^. Hitherto, molecular characterization of murine- and patient-derived organoids (PDO) for the identification of novel therapeutic targets has largely relied on genomic and transcriptomic profiling^4,7^, which often fails to accurately identify changes in enzymatic status or activity. Many proteins such as proteases, kinases, and metabolic enzymes are often regulated at the post-translational level by protein modifications such as ubiquitylation and phosphorylation. To accurately measure enzyme activities, the field of activity-based proteomics has gained significant attention in recent years with the use of small molecules, termed activity-based probes (ABPs)^9^. ABPs allow for covalent linkage in active-site residues of enzymes, which facilitates analysis of systems-wide changes at the level of enzyme activity rather than simple protein abundance. Mechanism-based ABPs have been developed to target different classes of enzymatic activities, including ubiquitin-conjugated ABPs (ubiquitin-ABPs) to identify deubiquitinating enzymes (DUBs)^10–12^. DUBs play an essential role in the ubiquitin-proteasome system by hydrolyzing the amide bond between mono- and poly-ubiquitin chains from substrate proteins^12–14^. DUBs therefore counteract the activity of ubiquitylating enzymes, thereby regulating the activity, stability, localization, and interactions of many substrate proteins.

The human genome encodes some 100 DUBs, of which five sub-types are characterized as cysteine peptidases comprising USPs (ubiquitin-specific proteases), UCHs (ubiquitin carboxy-terminal hydrolases), MJDs (Machado–Josephin domain-containing proteases), OTUs (ovarian tumor proteases) and MINDYs (motif-interacting with ubiquitin-containing novel DUB family). The well-defined catalytic cysteine residue in the active site of these enzymes, as well as their high degree of target selectivity, has made DUBs a promising therapeutic target in cancer^15–17^. However, their biological role and therapeutic potential have not yet been explored in PDAC.

In this study, we used an integrative approach combining PDOs and genetically modified mouse models with activity-based proteomics to identify DUBs active in PDAC. The functional significance of DUBs was assayed by a loss-of-function genetic screen in PDAC organoids which identified USP25 as an essential DUB required for PDAC tumor growth and viability. We found that USP25 is a master regulator of glycolysis by modulating hypoxia-inducible factor-1 alpha (HIF-1α) stability and transcriptional activity. Finally, pharmacological inhibition of USP25 in vitro and in vivo caused PDAC cell death and tumor regression, suggesting that USP25 is a promising therapeutic target in PDAC.

## Results

### Characterization of enzymatically active DUBs in PDAC using activity-based proteomics

**Fig. 1:**
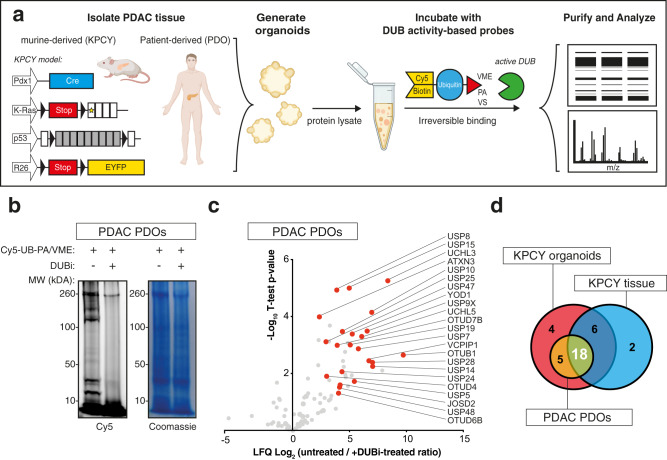
Characterization of PDAC specific DUBs using activity-based-probe proteomics. **a** Schematic overview of ABP experiment performed in PDAC organoids, created with BioRender.com. **b** In-gel visualization of Cy5-Ub-ABP labeled DUBs in patient-derived organoids (PDO). **c** Mass spectrometry analysis of DUBs labeled with Biotin-ubiquitin-ABP in PDOs. Volcano blot depicts the label-free quantification (LFQ) of −log_10_ t-test p-value vs ratio difference of untreated (right side) and DUBi-treated (left side) samples. Gray dots indicate all proteins identified, and red dots highlight all significant DUBs. **d** Venn diagram summarizing identified DUBs, from mass spectrometry experiments comparing untreated vs DUBi-treated samples. Source data are provided as a Source Date file.

To identify enzymatically active DUBs in PDAC, we developed an analysis pipeline combining ubiquitin-ABPs with mass spectrometry (Fig. 1a). To capture the maximum diversity of DUBs we pooled three classes of ABPs that included a mono-ubiquitin recognition element with either a propargylamide (PA), a vinylmethylester (VME) or a vinylsulfone (VS) electrophilic warhead that covalently interacts with the nucleophilic cysteine residue in the active sites of DUBs^10,11,18^. This allowed us to determine which DUBs are abundant and enzymatically active in three clinically relevant PDAC models: 1. pancreatic tumors from the widely used murine “KPCY” model, driven by oncogenic *Kras*^*G12D*^ mutation and inactivation of both alleles of *Trp53* plus a YFP tracer (KPCY mice: Pdx1-Cre; LSL-Kras^G12D^; Trp53^flox/flox^; Rosa26-LSL-YFP); 2. murine PDAC organoids (termed KPCY organoids) derived from advanced PDAC tumors from KPCY mice; and 3. patient-derived organoids (termed PDOs) derived directly from surgically resected PDAC tumors with their respective oncogenic driver mutations determined by sequencing (Fig. 1a). In-gel visualization of DUBs was achieved by incubation with the far-red fluorescent Cy5-conjugated ABPs, and both KPCY organoids and PDOs displayed strong Cy5 signal for proteins with molecular weights above 100 kDa and below 40 kDa, which largely corresponds to DUBs from the USPs and UCHs families, respectively (Fig. 1b, Supplementary Fig. 1a). Specificity of the probes for active DUBs was confirmed by co-treatment with the pan-DUB inhibitor PR-619 (referred to as DUBi)^19^. Co-treatment with DUBi attenuated DUB binding to the probes but not total protein abundance, as demonstrated by a reduction in Cy5 signal and equal Coomassie blue staining (Fig. 1b, Supplementary Fig. 1a). To elucidate the identities of these DUBs, cell lysates from KPCY organoids and PDOs were incubated with biotin-conjugated ABPs. Following label-free quantification of untreated and DUBi-treated samples, we identified 28 and 23 significantly active DUBs in KPCY organoids and PDOs, respectively (Fig. 1c, Supplementary Fig. 1b). Unbiased pathway analysis revealed that protein deubiquitination was the biological process most associated with the proteins interacting with the probes (Supplementary Fig. 1c, d), indicating that the ubiquitin-ABPs were specific for capturing DUBs. To verify the activity of DUBs in primary PDAC tumors, we isolated whole tumors from KPCY mice and labeled them with biotin-conjugated ABPs. Upon median normalization of the biological replicates, we again observed that ubiquitin-ABPs specifically enriches for DUBs in complex tissue lysates (Supplementary Fig. 1e). A total of 26 DUBs were significantly enriched in KPCY tumors (Supplementary Fig. 1e), 18 of which were also significantly enriched in both KPCY organoids and PDOs (Fig. 1d).

### Loss-of-function genetic screen identifies Usp25 as an essential DUB in PDAC organoids

**Fig. 2:**
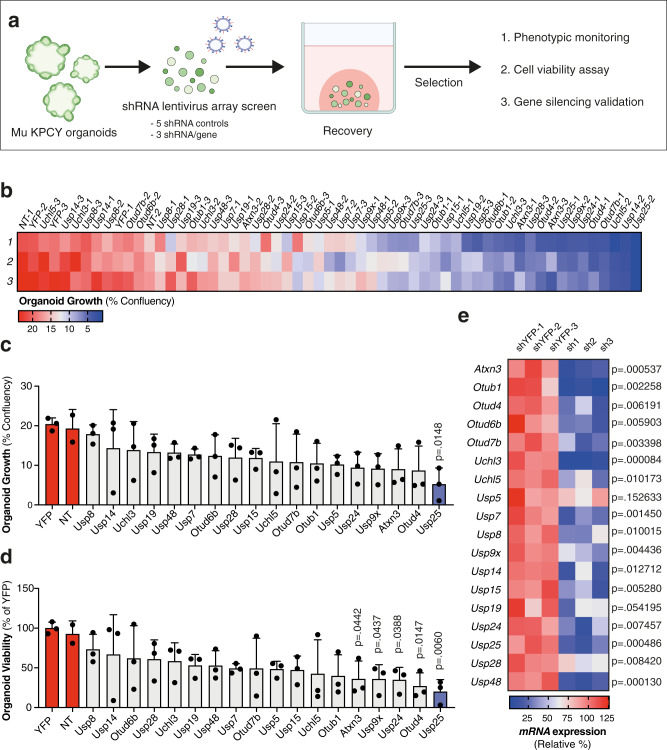
Genetic knock-down screen identifies USP25 as an essential DUB in PDAC organoids. **a** Schematic of genetic knock-down screen in KPCY organoids, created with BioRender.com. **b** Heatmap of percentage whole well growth confluency (% confluency) as measured by a high-content imaging system, 78 h post-puromycin selection. Biological replicates are shown for each individual shRNA target. **c** Organoid growth shown as % confluency, displayed as mean ± standard deviation (SD). Individual shRNA (black circles) values are shown for each gene, averaged from three biological replicates. Statistical significance was determined by One-way ANOVA with Dunnett post-hoc test (*n* = 3 biologically independent samples). **d** Organoid viability displayed as % relative to control shYFPs (red bars), displayed as mean ± SD. Individual shRNAs (black circles) are shown for each gene, averaged from three biological replicates. Statistical significance was determined by One-way ANOVA with Dunnett post-hoc test (*n* = 3 biologically independent samples). **e** Heatmap of mRNA expression by qPCR for each individual shRNA per gene, relative to the average of three independent shYFPs. Statistical significance was determined by two-tailed Student’s *t* test with Holm–Sidak post-hoc correction for multiple testing (*n* = 3 biologically independent samples). Source data are provided as a Source Date file.

To investigate the function of the 18 most highly active DUBs, we performed a loss-of-function genetic screen in PDAC organoids (Fig. 2a). We designed a lentivirus shRNA array library in the pLKO.1 vector system, containing three validated shRNAs per gene as well as five different control shRNAs. Since we observed that DUB activity is highly conserved amongst KPCY organoids and PDOs, we opted to use KPCY organoids as they are genetically defined, display uniform morphology and exhibit a reproducible growth rate in our high-throughput assay conditions. In addition, these organoids express a YFP fluorescent protein driven by the Rosa26 promoter, which we used as an shRNA control (shYFP). When compared with organoids transduced with non-targeting shRNA (referred to as shNT), organoids treated with YFP-targeting hairpins showed reduced YFP expression following selection (Supplementary Fig. 2a, b), but organoid growth and viability were unaffected (Supplementary Fig. 2c, d). We next assessed organoid growth over time using high-content imaging, and observed that KPCY organoid sphere formation and growth reached its peak between 72–90 h post-seeding (Supplementary Fig. 2e, f). Following transduction with the DUB shRNA library and phenotypic monitoring post-selection, hierarchical clustering revealed that several DUB-targeting shRNAs diminished organoid growth compared with controls in independent experiments (Fig. 2b), with *Usp25* depletion giving the most pronounced inhibition (Fig. 2c, d). All three *Usp25* targeting shRNAs significantly diminished organoid growth and viability (Fig. 2c, d), and this was correlated with a reduction in *Usp25* gene expression (Fig. 2e).

### USP25 is highly expressed and enzymatically active in PDAC compared to normal pancreatic tissue, which correlated with poorer patient survival

**Fig. 3:**
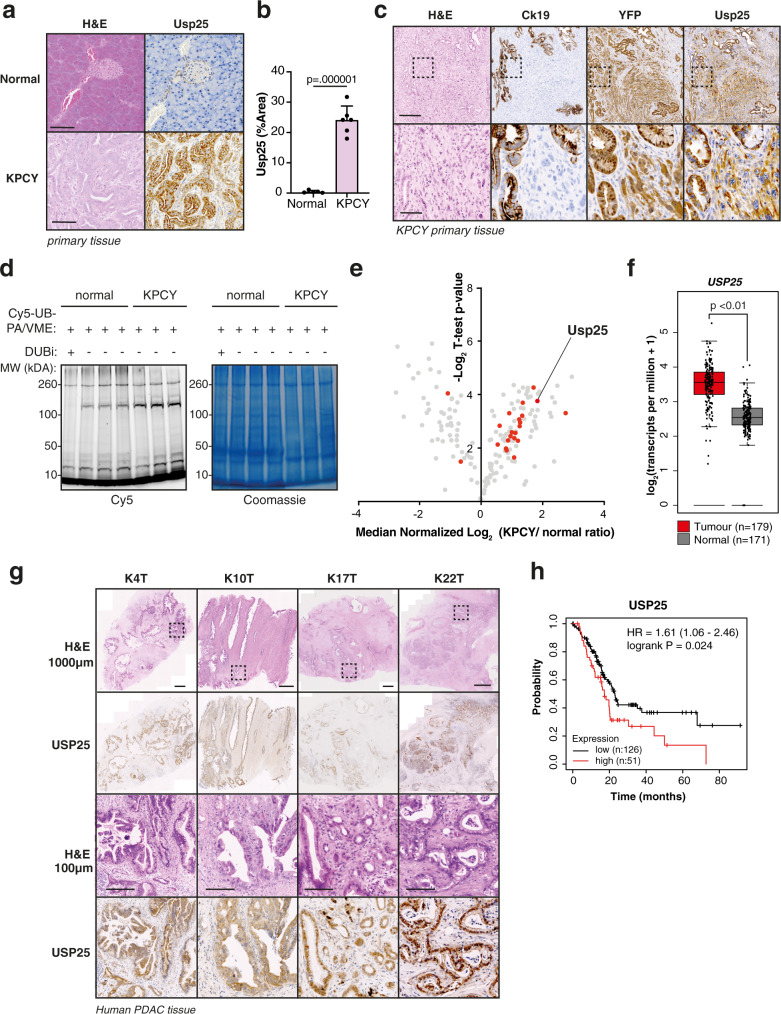
USP25 is highly expressed and enzymatically active in PDAC compared to normal pancreatic tissue, which correlated with poor patient survival. **a** Histological and immunohistochemical analysis of Usp25 expression in normal pancreatic and KPCY tumor tissues. Scale bar is 100 µm. **b** Quantification of Usp25 positive staining area (*n* = 6 biologically independent animals per group), displayed as mean ± SD. Statistical significance was determined by two-tailed Student’s *t* test. **c** Histological and immunohistochemical analysis of Usp25 expression in KPCY tumor tissue (*n* = 5 biologically independent animals per group). Scale bar is 1000 µm, insert is 100 µm. **d** In-gel visualization of Cy5-Ub-ABP labeled DUBs in murine normal pancreatic vs KPCY tumor tissues (*n* = 3 biologically independent animals per group). **e** Mass spectrometry analysis of DUBs labeled with Biotin-ubiquitin-ABP in normal pancreatic and KPCY tumor tissue. Biological replicates were averaged and displayed as median normalized −log_10_ t-test *p*-value vs ratio difference of normal (left side) and KPCY tumor (right side) samples, and red dots highlight all significant DUBs. **f** *USP25* expression in PDAC tumor and normal tissues based on publicly available TCGA and GTEx RNA-seq datasets, generated using GEPIA online platform, with the parameters log2fold-change cutoff 1 and *p*-value cutoff 0.01. In box plot, the center line reflects the median. **g** Histological and immunohistochemical analysis of USP25 staining in primary patient PDAC tumors. Scale bar is 100 µm. **h** Kaplan–Meier survival plot of PDAC patients expressing above (*n* = 51, red) or below (*n* = 126, black) levels of *USP25*, generated using the Kaplan-Meier plotter online platform. Vertical ticks represent censored events; logrank *p* = 0.024, two-tailed log-rank test. Source data are provided as a Source Date file.

To evaluate the role of Usp25 in PDAC, we first compared its expression and enzymatic activity in PDAC with that in healthy pancreatic tissue. We observed a significant upregulation of Usp25 protein expression in the KPCY model, compared with normal pancreatic tissue (Fig. 3a, b, Supplementary Fig. 3a) Immunohistochemistry (IHC) revealed strong cytosolic Usp25 staining in transformed KPCY pancreas, which overlapped with the YFP lineage tracer (Fig. 3c). Usp25 was expressed in both epithelial PDAC cells, which co-stained with CK-19, and in poorly differentiated mesenchymal PDAC regions (Fig. 3c). Interestingly, we observed that Usp25 is absent in the surrounding stroma cells, indicating that Usp25 is expressed specifically in tumor cells. To determine if Usp25 upregulation leads to increased enzymatic activity, we labeled KPCY and normal pancreatic tissues with ubiquitin-ABPs and observed that many DUBs, including Usp25, are significantly more active in KPCY tumors (Fig. 3d, e). We next assessed the role of USP25 in human PDAC and found that *USP25* messenger RNA was highly expressed in human PDAC relative to normal pancreatic tissue (Fig. 3f). Moreover, we detected strong IHC staining of USP25 in a panel of PDAC patient samples (Fig. 3g, Supplementary Fig. 3b). Analysis of publicly available datasets revealed that high *USP25* messenger RNA expression is associated with shorter overall survival (*n* = 177, Fig. 3h). Together, these findings indicate that USP25 may play an important role in PDAC progression.

### Depletion of *USP25* leads to reduced patient-derived organoid viability, survival and attenuated PDAC tumor growth in vivo

**Fig. 4:**
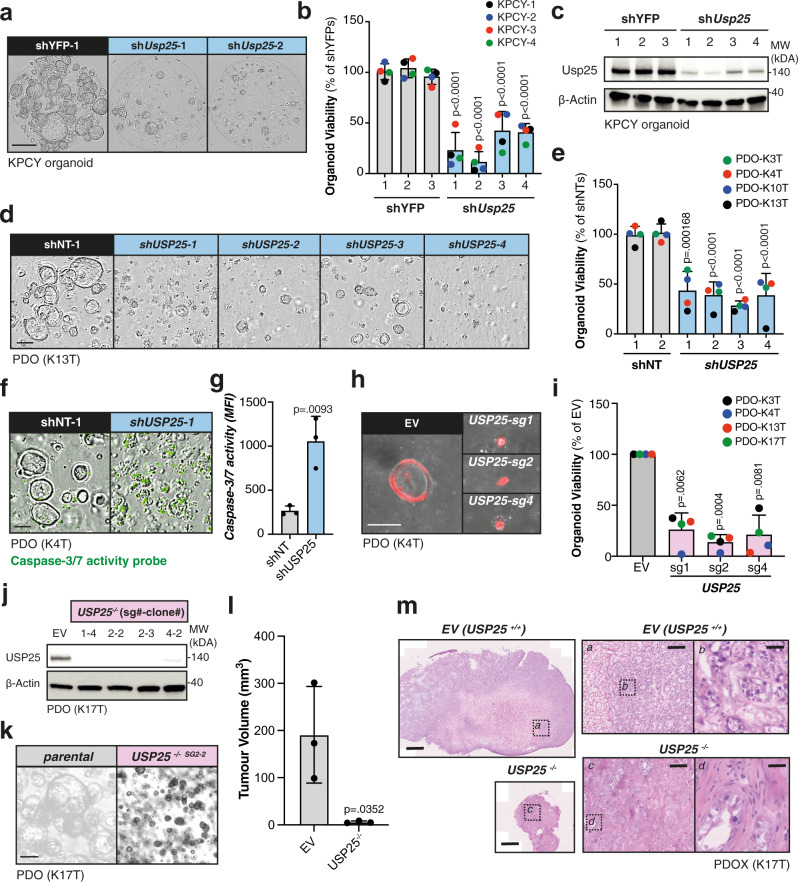
Depletion of *USP25* leads to reduced patient-derived organoid formation, viability and attenuated PDAC tumor growth in vivo. **a** Representative images of sh*Usp25* silencing in KPCY organoids. Scale bar is 800 µm. **b** KPCY organoid viability displayed as % relative to shYFP controls (*n* = 4 biologically independent samples). **c** Western blot of endogenous Usp25 in KPCY organoids. **d** Representative images of sh*USP25* silencing in PDO lines. Scale bar is 800 µm. **e** PDO viability displayed as % relative to shNT controls (*n* = 4 biologically independent samples). **f** Representative images of PDO lines incubated with caspase-3 activity probe. Scale bar is 800 µm. **g** Quantification of caspase-3 signal in (**f**), shown as mean fluorescent intensity (MFI). Each symbol represents a different shRNA targeting either *USP25* or NT control, averaged from biologically independent experiments (*n* = 3). **h** Representative images of the PDO lines (K4T), showing empty vector (EV) control and CRISPR/Cas9-mediated *USP25* knock-out cells. Each *USP25*^*−/−*^ clones are described with single guide (sg) targeting exon shown. Scale bar is 400 µm. **i** Organoid viability displayed as % relative to the EV. **j** Western blot of endogenous USP25. **k** Representative images of PDO parental and USP25^−/−^ clones. Scale bar is 400 µm. **l** PDOX tumor volumes at end point. **m** Histological analysis of PDOX at end point. Scale bar is 1000 µm, inserts are 100 µm and 50 µm. Bar graphs in (**b**, **e**, **g**, **i**, **l**) are displayed as mean ± SD, and each colored circle in (**b**, **e**, **i**) represents a different organoid line, derived from an independent animal or patient. Statistical significance in (**g**, **l**) was determined by two-tailed Student’s *t* test, and in (**b**, **e**, **i**) by One-way ANOVA with Dunnett post-hoc test. Source data are provided as a Source Date file.

We next assessed the biological role of Usp25 by silencing its expression in KPCY organoids. Four independent *Usp25*-targeting shRNAs all induced a significant reduction in organoid formation and growth (Fig. 4a, Supplementary Fig. 4a). This reduction in organoid formation led to a 71% overall reduction in organoid viability relative to organoids transduced with control shRNAs (Fig. 4b), which correlated with *Usp25* knock-down efficiency (Fig. 4c, Supplementary Fig. 4b). A similar phenotype was observed with shRNA-mediated knock-down of *USP25* in four different PDO lines (Fig. 4d, e). Again, we found that loss of organoid viability correlated with *USP25* silencing efficiency (Supplementary Fig. 4c). To uncover the mechanism behind this loss of growth and viability we incubated shRNA-transduced PDOs with a cell–permeable caspase-3 activity probe to measure apoptosis-mediated cell death. Following *USP25* knock-down, we detected significant caspase-3 activity (Fig. 4f, g, Supplementary Fig. 4d), indicative of apoptosis.

We then generated multiple PDO lines in which the *USP25* gene was homozygous ablated using CRISPR/Cas9 using three independent single guide RNAs (sgRNAs) (Supplementary Fig. 4e). We observed significantly reduced organoid formation and an 80% overall reduction in viability upon *USP25* knock-out in four independent PDO lines compared to empty vector (EV) control organoids (Fig. 4h, i). Following clonal expansion of surviving organoids, we were unable to generate viable *USP25*^*−/−*^ clones from many of the PDO lines, except line K17T, which presented frame-shift mutations at the targeted exons and loss of USP25 protein expression (Fig. 4j, Supplementary Fig. 4f). Interestingly, we observed that *USP25*^*−/−*^ organoids grew as small, solid clusters with reduced viability compared to EV control organoids (Fig. 4k, Supplementary Fig. 4g, h). Deletion of *USP25* not only impaired PDO growth in vitro, but led to a dramatic loss of patient-derived organoid xenograft (PDOX) growth in vivo (Fig. 4l). In addition, *USP25* knock-out PDOXs displayed impaired tumor heterogeneity, composed mainly of stroma cells with very few tumor cells (Fig. 4m). Collectively, these findings demonstrate that USP25 plays an essential role in PDAC growth and maintenance.

### Transcriptional and metabolomic profiling of PDAC organoids reveals USP25 as a novel regulator of HIF-1 transcriptional activity and metabolic rewiring

**Fig. 5:**
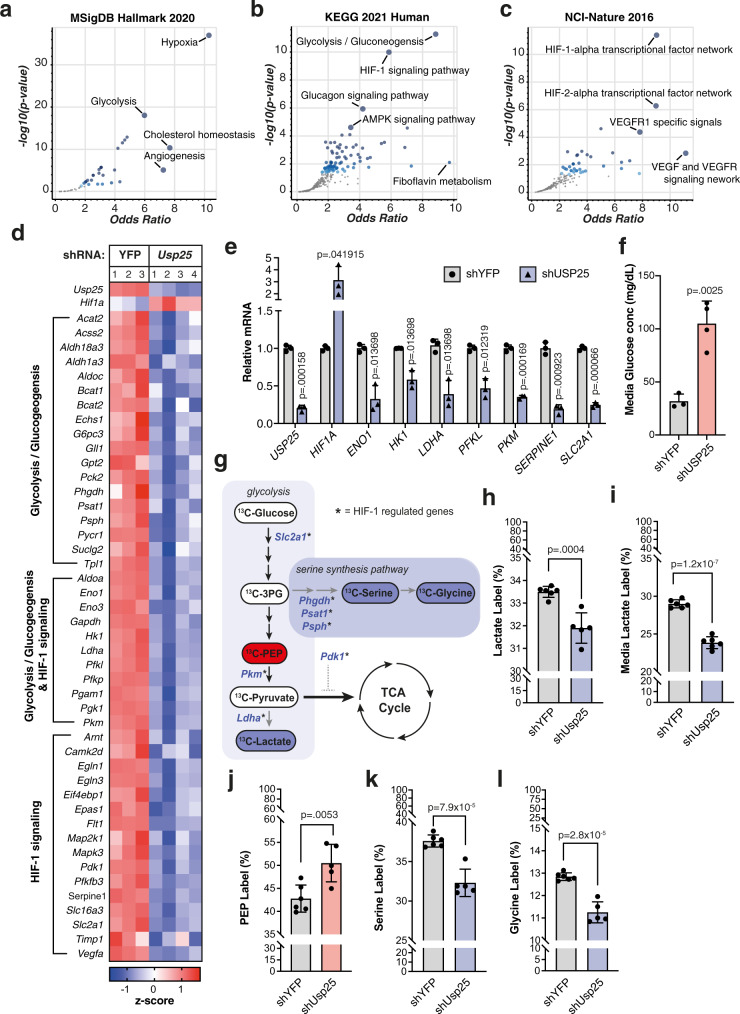
Transcriptional and metabolomic profiling of PDAC organoids reveals USP25 as a novel regulator of HIF-1 transcriptional activity and metabolic rewiring. **a**–**c** Pathway analysis from (**a**) MSigDB Hallmark 2020, (**b**) KEGG 2021 Human, and (**c**) NCI-Nature 2016 datasets of differentially expressed genes downregulated upon loss of *Usp25*. The volcano plot shows the significance of each gene set from the selected library versus its odds ratio. Larger blue points represent significant terms (*p*-value < 0.05); smaller gray points represent non-significant terms. The darker the blue color of a point, the more significant it is. **d** Heatmap of gene expression z-scores for top KEGG pathways. **e** Gene expression in PDOs displayed as relative mRNA expression compared to shYFP controls. Each symbol represents independent shRNAs targeting either *YFP* (black circles) or *USP25* (black triangles), averaged from biologically independent experiments (*n* = 3), and displayed as mean ± SD. Statistical significance was determined by two-tailed Student’s *t* test with Holm–Sidak post-hoc correction for multiple testing. **f** Glucose concentrations measured in culture medium from PDO (*n* = 3 biologically independent experiments), displayed as mean ± SD. Statistical significance was determined by two-tailed Student’s *t* test. **g** Schematic of ^13^C-glucose enrichment analysis in KPCY organoids treated with sh*Usp25*, with downregulated HIF-1 target genes in blue and measured metabolites that are unchanged (white), downregulated (blue) or enriched (red) compared to shYFP-treated organoids. **h**–**l** Incorporation of ^13^C-glucose into (**h**) intracellular lactate, (**i**) secreted lactate in the culture media, (**j**) intracellular PEP, (**k**) intracellular serine, and (**l**) intracellular glycine in treated KPCY organoids. Data in (**h**–**l**) is displayed as mean ± SD, and represents one experiment carried out with six replicates. Due to technical limitations, statistics for (**h**–**l**) were performed on technical replicates using two-tailed Student’s *t* test. Source data are provided as a Source Date file.

To understand the molecular mechanisms underlying PDAC vulnerability induced by *Usp25/USP25* depletion, we performed RNA sequencing (RNA-seq) on KPCY organoids after shRNA silencing but before significant changes in viability were observed. Hierarchical clustering and principal component analysis (PCA) revealed distinct gene expression profiles when comparing sh*Usp25* and shYFP control groups (Supplementary Fig. 5a, b). Statistical analysis showed that 1880 protein-coding transcripts were differentially expressed (FDR adjusted *p*-value ≤0.05 and Log_2_ fold-change −0.5> *x* >0.5) between the two groups. Pathway analysis revealed that transcripts significantly downregulated upon *Usp25* silencing were highly enriched for genes involved in hypoxia, glycolysis, and HIF-1 activity pathways (Fig. 5a–c, Supplementary Fig. 5c–d).

One defining feature of human PDAC is its severely hypoxic microenvironment^20,21^. During hypoxia, the transcription factor hypoxia-inducible factor-1 alpha (HIF-1α) is stabilized and induces the expression of genes involved in glycolysis and hypoxia. High expression levels of HIF-1α and its target genes have been described to promote tumor aggressiveness, chemoresistance, and poor overall patient survival in PDAC^22–27^. Interestingly, we found that *USP25* expression is positively correlated with both glycolysis and hypoxia gene signatures in PDAC patients (Supplementary Fig. 5e, f). Analysis of transcripts involved in the hypoxia, glycolysis/gluconeogenesis, and HIF-1 signaling pathways showed a significant reduction in expression of these genes upon *Usp25* depletion (Fig. 5d, Supplementary Fig. 6a). The expression of canonical HIF-1α target genes decreased in both KPCY organoids and PDOs upon *Usp25/USP25* silencing (Fig. 5e, Supplementary Fig. 6b). Concomitant with reduced expression of key enzymes involved in glycolysis, expression of the glucose transporter *SLC2A1* (also referred to as GLUT1) was also reduced upon *Usp25*/*USP25* depletion. This was accompanied by a substantial decrease in glucose uptake from the culture medium over time (Fig. 5f, Supplementary Fig. 6c).

To understand how loss of HIF-1α target genes impacted cellular glucose metabolism, we performed an isotopic labeling enrichment analysis using ^13^C-glucose (Fig. 5g). We observed a rewiring of glycolysis upon *Usp25* silencing (Fig. 5g), characterized by a significant reduction in both lactate production and de novo lactate secretion (Fig. 5h, i). In addition, *Usp25* depletion led to a significant enrichment of glucose-derived carbon into phosphoenolpyruvate (PEP) (Fig. 5j) and a substantial decrease in de novo serine and glycine synthesis (Fig. 5k, l). These metabolic changes were consistent with reductions in the HIF-1α regulated enzymes *Phgdh*, *Psat1, Psph, Pkm* and *Ldha* observed in our RNA-seq dataset (Fig. 5d). Notably, these changes were restricted to glycolysis and glycolytic shunt pathways, as no significant changes were observed in labeled pyruvate or TCA cycle metabolites (Supplementary Fig. 6d, e). These results suggest that USP25 is required for HIF-1-mediated metabolic rewiring in PDAC (Fig. 5g).

### USP25 interacts with, deubiquitinates and stabilizes HIF-1α and promotes its transcriptional activity

**Fig. 6:**
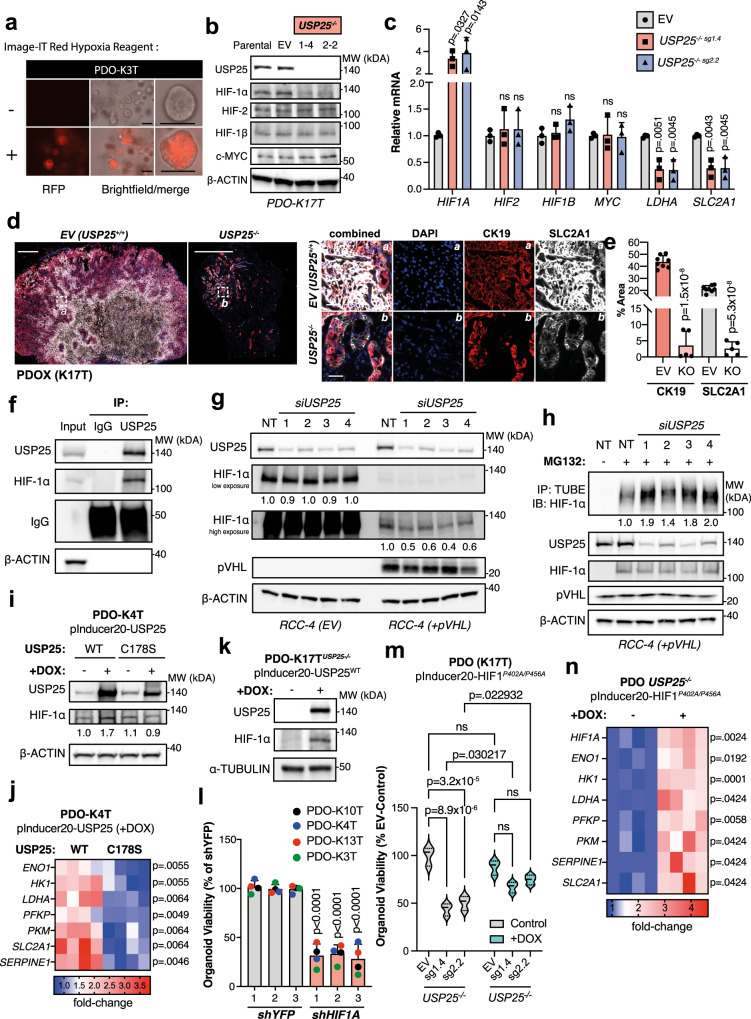
USP25 interacts with, deubiquitinates and stabilizes HIF-1α and promotes its transcriptional activity. **a** PDOs (line K3T) were treated with the cell-permeable Image-IT Red Hypoxia reagent. Scale bar is 100 µm. **b** Western blot of endogenous protein levels in parental, empty vector (EV) and USP25^−/−^ clone PDO lines (line K17T). **c** Gene expression in USP25^−/−^ PDO lines displayed as relative mRNA expression compared to EV PDO lines, shown as mean ± SD. Statistical significance was determined by One-way ANOVA with Dunnett post-hoc test (*n* = 3 biologically independent experiments). **d** Representative images of immunofluorescent staining from PDOX of SLC2A1 (gray) CK19 (red), and DAPI (blue). Scale bar is 1000 µm, insert is 50 µm. **e** Quantification of staining % Area of CK19 and SLC2A1 displayed as mean ± SD. Statistical significance was determined by two-tailed Student’s *t* test (*n* = 7 independent animals in EV and *n* = 5 independent animals in KO group). **f** Co-immunoprecipitated endogenous USP25 and immunoblotted targets as indicated in HEK293T cells. **g** Western blot of indicated targets following siRNA-mediated knock-down of *USP25* or non-targeting (NT) control. **h** siRNA-mediated knock-down of *USP25* or NT control cells treated with 25 µM MG132 for 8 h. Lysates were incubated with tandem ubiquitin binding entities (TUBE) coupled agarose beads and immunoblotted as indicated. **i** Western blot images of endogenous protein levels following doxycycline (DOX) treatment for induction of USP25 wild-type (WT) or catalytic mutant (C178S) expression. **j** Heatmap of gene expression after DOX-induced expression, shown as relative fold-change to USP25^C178S^. Statistical significance was determined by two-tailed Student’s *t* test with Holm–Sidak post-hoc correction for multiple testing (*n* = 4 biologically independent experiments). **k** Western blot images of endogenous protein levels following DOX treatment in USP25^−/−^ PDOs for expression of USP25 wild-type. **l** Organoid viability displayed as % relative to controls (shYFP, gray bars), shown as mean ± SD. Statistical significance was determined by One-way ANOVA with Dunnett post-hoc test (*n* = 4 biologically independent experiments). **m** Violin plot of organoid viability displayed as % relative to EV untreated controls after DOX-induced expression of the HIF-1α stabilization mutant (HIF1A^P402A/P456A^). Statistical testing was done with two-way ANOVA with a multiple comparison correction test (*n* = 3 biologically independent experiment). **n** Heatmap of gene expression after DOX-induced expression. Data shown as relative fold-change to untreated control. Statistical significance was determined by two-tailed Student’s *t* test with Holm–Sidak post-hoc correction for multiple testing (*n* = 4 biologically independent experiments). Source data are provided as a Source Date file.

To elucidate the link between USP25 and HIF-1α in more detail, we measured hypoxia and HIF-1α protein levels in multiple human PDAC cell lines and PDOs. In human PDAC cell lines grown as a 2D monolayer, HIF-1α protein was reduced upon U*SP25* silencing when treated with a hypoxia-mimetic compound (Supplementary Fig. 7a). Interestingly, PDOs cultured in 3D Matrigel were intrinsically hypoxic under normoxic conditions (Fig. 6a, Supplementary Fig. 7b), and were more sensitive to *USP25* depletion when cultured under low oxygen (1% O_2_) tension (Supplementary Fig. 7c). Moreover, HIF-1α protein was readily detectable in PDOs (Fig. 6b, Supplementary Fig. 7d) and significantly reduced upon *USP25* silencing in PDOs (Supplementary Fig. 7d). These changes were specific to HIF-1α, as we did not observe changes in the expression of other HIF isoforms (i.e. HIF-2 and HIF-1β) (Fig. 6b, c). In addition, we did not detect changes in the transcription factor c-MYC upon *USP25* depletion (Fig. 6b, c), which has previously been linked to HIF activity and tumor metabolism^28–31^. Furthermore, the decrease in HIF-1α protein abundance occurred post-translationally, as *USP25* deletion led to an increase in *HIF-1α* transcript expression and a significant down-regulation of its transcriptional targets *LDHA* and *SLC2A1* (Fig. 6c). These effects were mirrored in vivo, as PDOX tumors lacking *USP25* showed diminished SLC2A1 expression in CK19-positive tumor cells (Fig. 6d, e). Collectively, these findings suggest that USP25 regulates HIF-1α protein abundance in a post-translational manner.

We next investigated if USP25 directly interacts with HIF-1α by preforming co-immunoprecipitation experiments. Using ectopically expressed HIF-1α and USP25, we detected a strong interaction between the two proteins under normoxic and hypoxia-mimetic conditions (Supplementary Fig. 8a). Likewise, endogenous USP25 co-immunoprecipitated with endogenous HIF-1α (Fig. 6f). To investigate this regulation further we turned to well-described cellular model systems. Under normoxia, HIF-1α is expressed but continuously degraded by the von Hippel-Lindau tumor suppressor gene product (pVHL)-mediated ubiquitin-proteasomal degradation^32,33^. Under normoxic conditions, constitutively high protein levels of HIF-1α in the pVHL-deficient renal carcinoma cell line RCC-4 were unaffected by depletion of *USP25* (Supplementary Fig. 8b). However, when the pVHL gene was reintroduced, we observed a significant decrease in HIF-1α protein levels that was further reduced upon *USP25* silencing (Fig. 6g). Consistent with earlier findings (Fig. 6b), we did not detect changes in other HIF isoforms, HIF-2 and HIF-1β (Supplementary Fig. 8c). These data suggest that USP25 specifically counteracts pVHL-mediated ubiquitylation of HIF-1α. Treatment of pVHL-expressing RCC-4 cells with the proteasomal inhibitor MG132 resulted in the accumulation of ubiquitylated HIF-1α (Fig. 6h). Upon silencing of *USP25* we observed an increase in ubiquitylated HIF-1α, suggesting that USP25 deubiquitinates HIF-1α (Fig. 6h), thereby antagonizing pVHL-mediated ubiquitylation.

We next examined the effect of USP25 gain-of-function on HIF-1α stability and nuclear translocation. Ectopic expression of wild-type USP25 (USP25^WT^, or WT), but not of a catalytically inactive USP25 mutant (USP25^C178S^, or CS) led to a substantial increase in the stability of both cytoplasmic and nuclear HIF-1α, which was further enhanced under hypoxia-mimetic conditions (Supplementary Fig. 8d). This increase in nuclear HIF-1α led to a significant upregulation in the expression of its transcriptional target gene *SLC2A1* (Supplementary Fig. 8e). These data indicate that a catalytically-active USP25 is needed for HIF-1α stability and transcriptional activity. We confirmed these findings in several PDO lines, by engineering doxycycline-inducible lines with either USP25^WT^ or USP25^C178S^ expression (Supplementary Fig. 8f). Upon doxycycline treatment we detected robust upregulation of USP25 expression and found that only wild-type USP25 could increase HIF-1α protein levels (Fig. 6i, Supplementary Fig. 8g) and transcription of its target genes (Fig. 6j). Importantly, reintroducing USP25 into *USP25*-depleted PDOs rescued HIF-1α protein expression (Fig. 6k). In addition, silencing *HIF1A* expression recapitulated the effects of *USP25* loss on PDO growth and viability, as well as down-regulation of key glycolytic enzymes (Fig. 6l and Supplementary Fig. 8h). On the other hand, reintroducing a stabilized HIF-1α mutant (HA-tagged HIF1A^P402A/P456A^) into *USP25*-depleted PDO lines (Supplementary Fig. 8i) partially rescued both organoid viability (Fig. 6m) and glycolytic target gene expression (Fig. 6n). Collectively, these findings suggest that PDAC tumors require USP25 function to promote HIF-1α stability and transcriptional activity.

### Pharmacological inhibition of USP25 leads to loss of HIF-1α signaling and reduced tumor growth in vitro and in vivo

**Fig. 7:**
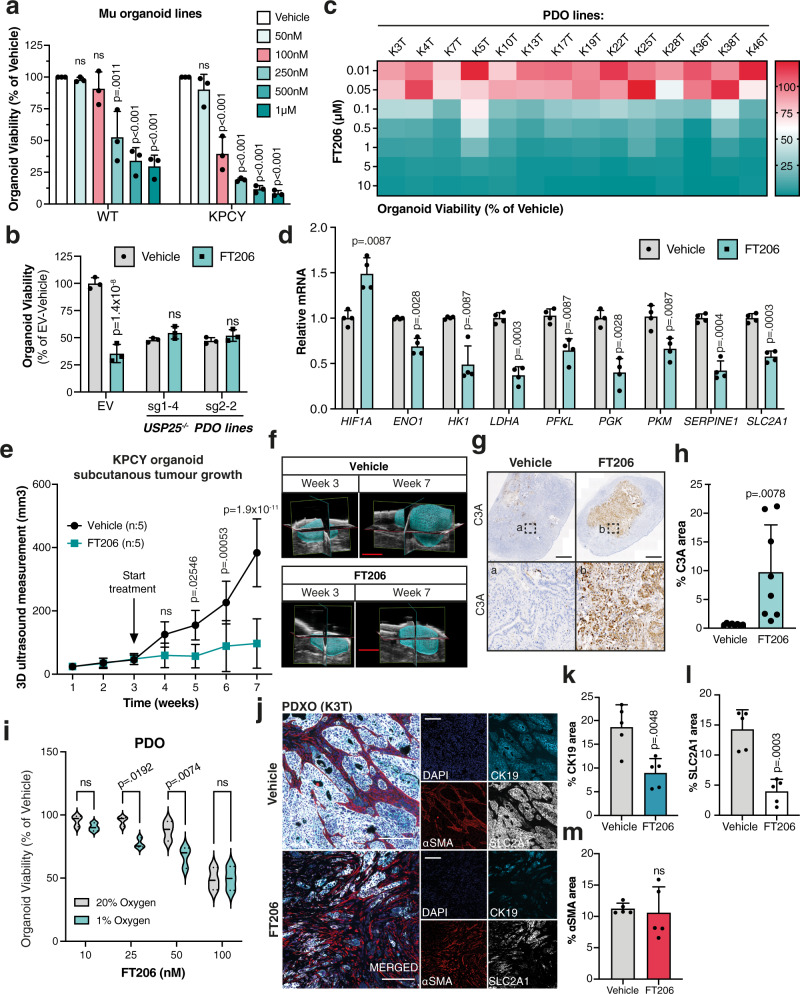
Pharmacological inhibition of USP25 leads to loss of HIF-1α signaling and reduced tumor growth in vitro and in vivo. **a** Organoids treated with FT206 at the indicated doses for 72 h. Organoid viability was displayed as % relative to vehicle treated control. Statistical significance was determined by One-way ANOVA with Dunnett post-hoc test (n = 3 biologically independent experiments). **b** Organoid viability after 100 nM FT206 treatment, and displayed as % relative to EV vehicle treated. Statistical significance was determined by Two-way ANOVA with post-hoc correction for multiple testing (*n* = 3 biologically independent experiments). **c** Heatmap displaying organoid viability of PDO lines treated FT206 with indicated concentrations, calculated as % of vehicle treated. **d** Gene expression in PDO lines, displayed as relative mRNA expression compared to vehicle-treated control. Statistical significance was determined by Student’s *t* test with Holm–Sidak post-hoc correction for multiple testing (*n* = 4 biologically independent experiments). **e** Growth curves of subcutaneous tumor volume (mm^3^) from KPCY organoids, treated with vehicle or FT206 (*n* = 5 independent animals per group). **f** Representative 3D ultrasound images of subcutaneous tumors described in (**e**). Scale bar is 3 mm. **g** Representative immunohistochemical analysis of active cleaved caspase-3 (C3A) expression in subcutaneous tumors. Scale bar is 1000 µm, insert is 100 µm. **h** Quantification of C3A staining (*n* = 8 independent animals per group). **i** PDO lines were grown in ether normoxic (20% Oxygen) or hypoxic (1% Oxygen) growing conditions and treated with the indicated concentration of FT206. Violin plot of organoid viability was displayed as % relative to vehicle treated (*n* = 3 biologically independent experiments). **j** Representative images of immunofluorescent staining for DAPI (dark blue), CK19 (light blue), αSMA (red) and SLC2A1 (white) in PDOXs treated with vehicle or FT206. Scale bar is 200 µm. **k**–**m** Quantification of positive staining area in whole PDOX sections (*n* = 5 independent animals per group) for (**k**) CK19, (**l**) SLC2A1, and (**m**) αSMA. Bar graphs in (**a**, **b**, **d**, **h**, **k**–**m**) represent the mean ± SD. Statistical significance in (**h**, **k**, **l**, **m**) was determined by two-tailed Student’s *t* test. Statistical significance in (**b**, **e**, **i**) was determined by Two-way ANOVA with post-hoc correction for multiple testing. Source data are provided as a Source Date file.

To investigate the therapeutic potential of targeting USP25 in PDAC, we used a novel pre-clinical pharmacological inhibitor of USP25, termed FT206^34^. This compound inhibits both USP25 and USP28, which share 57% amino acid sequence identity in their catalytic domains^35^. Interestingly, whereas growth and viability of KPCY organoids were sensitive to FT206 treatment with an IC_50_ concentration of 100 nM, normal pancreatic ductal organoids were unaffected at this concentration (Fig. 7a, Supplementary Fig. 9a). Moreover, sensitivity to FT206 was dependent on USP25 expression (Fig. 7b), and did not correlate with USP28 expression levels (Supplementary Fig. 9b, c). We next treated a panel of 13 unique PDO lines (Supplementary Table 1) and observed that almost all (12/13) lines were sensitive to 100 nM FT206 (Fig. 7c, Supplementary Fig. 9d). We next assessed if pharmacological inhibition of USP25 by FT206 mimics the effects of genetic silencing of *Usp25/USP25* on HIF-1α activity. In a panel of KPCY and PDO lines, treatment with FT206 led to a significant down-regulation of HIF-1α target genes (Fig. 7d and Supplementary Fig. 9e). USP25 has also previously been shown to deubiquitinate tankyrases, leading to upregulation of canonical Wnt-β-catenin signaling^36^. We therefore assessed the effects of tankyrase and Wnt signaling inhibition, which showed little effect on both KPCY organoids and PDO lines when compared to FT206 treatment (Supplementary Fig. 9f, g). Hence, pharmacological inhibition of USP25 decreases HIF-1α activity and attenuates PDAC organoid growth, which is not recapitulated by inhibition of other known USP25 substrates.

FT206 was previously shown to be well tolerated in mice^34^ so we next explored Usp25 as a therapeutic PDAC target in vivo using a subcutaneous transplantation PDAC model. Administration of FT206 in vivo triggered a profound growth inhibition in both KPCY organoid-derived tumors (Fig. 7e, f and Supplementary Fig. 10a) and in two different PDOX models (Supplementary Fig. 10c–e), without toxicity (Supplementary Fig. 10b, f). Interestingly, histological characterization showed that FT206 treatment led to a dramatic increase in apoptosis in the tumor core (Fig. 7g, h and Supplementary Fig. 10g). Consistent with the idea that tumor cores are more hypoxic, we observed that PDOs were more sensitive to FT206 treatment when grown under low oxygen tension (Fig. 7i). In vivo, the PDOX models recapitulated primary PDAC tumors by displaying high levels of hypoxia (Supplementary Fig. 11a, b), yet there was a significant reduction in CK19-positive tumor cells within these hypoxic PDOX tumors (Fig. 7j, k, Supplementary Fig. 11a). Moreover, histological characterization of HIF-1α target genes in both murine subcutaneous and PDOX tumors revealed a significant reduction in Slc2a1/SLC2A1 protein levels upon FT206 treatment (Fig. 7j, l, Supplementary Fig. 11c, d). Importantly, SLC2A1 co-stained with CK19 and was absent from alpha-smooth muscle actin (αSMA) positive stroma cells, suggesting that SLC2A1 is present predominantly in PDAC tumor cells (Fig. 7j–m). In PDAC patient samples we also detected strong IHC staining of SLC2A1 primarily in tumor cells, which was positively correlated with USP25 protein expression (Supplementary Fig. 11g, h). Of note, in subcutaneous tumors treated with FT206, we observed reduced angiogenesis and a measurable decrease in the endothelial cell marker Lectin (Supplementary Fig. 11c, e, f), consistent with reduced levels of HIF-1α activity. Collectively, these results show that pharmacological inhibition of USP25 leads to reduced HIF-1α activity specifically in PDAC tumor cells, which impairs their survival in the hypoxic tumor microenvironment, and subsequently leads to a reduction in PDAC tumor growth in vivo (Supplementary Fig. 12).

## Discussion

In this study, we used activity-based proteomics coupled with a genetic loss-of-function screen to identify DUBs required for PDAC maintenance and survival. We identified a novel dependency of PDAC on USP25. PDAC-derived organoids deficient in *Usp25/USP25* had dramatically impaired organoid growth and decreased viability. Mechanistically, we identified that PDAC-derived organoids were intrinsically hypoxic and required USP25 for regulation of HIF-1α protein stability, transcriptional activity, and glycolysis. Importantly, both genetic deletion and pharmacological inhibition of USP25 dramatically reduced the growth of PDOXs, highlighting the importance of the USP25-HIF-1α-glycolytic axis in PDAC maintenance.

So far, identifying novel therapeutic targets has largely relied on transcriptomic and proteomic approaches. These are powerful resources to determine when specific genes are expressed and translated, but often fail to show when an enzyme is active and performs its biological function. Many desirable drug targets are regulated at the posttranslational level, but the activity state of an enzyme cannot be inferred by measurements of mRNA or protein abundance. Activity-based proteomics circumvents these limitations and increases the efficiency of drug discovery when applied to clinically relevant human and murine genetic models. Our activity-based proteomics analysis revealed that 18 DUBs were highly active in PDOs, as well as in whole KPCY tumors, out of the 100 known DUBs in the human genome. Importantly, this allowed us to functionally validate a manageable number of candidate targets in primary PDAC organoids. Thus, activity-based proteomics offers a strategy to identify candidate enzymatic targets that are both expressed and biologically relevant in a specific cellular system or tissue, which can then be coupled with focused genetic and pharmacological validation studies. In this study, we concentrated on identifying active DUBs, which are viewed as a novel class of druggable enzymes in cancer^15–17^, but in the future different mechanism-based ABPs could be used to explore other classes of druggable enzymes in PDAC, such as kinases or phosphatases.

Two signature features of PDAC are altered glucose metabolism and a severely hypoxic microenvironment^21^. The transcription factor HIF-1α plays a major role in regulating these processes, and has been linked to tumor survival, progression, and chemotherapy resistance in PDAC^22–24,26,27^. However, recent studies have also suggested that *HIF1A* may function as a tumor suppressor during PDAC initiation^37,38^, highlighting the dual function of this protein. Our study identifies USP25 as a novel regulator of metabolic rewiring, maintenance, and survival of advanced PDAC through regulation of HIF-1α stability and transcriptional regulation. Moreover, these findings were directly dependent on the USP25-HIF-1α-axis, as USP25 did not regulate the stability or activity of other HIF isoforms. We also did not observe a compensation of c-MYC when targeting HIF-1α through USP25, which has previously been linked to HIF activity^28–31^ and shown to be upregulated in a PDAC initiation tumor model with *HIF1A* deletion^37^. Instead, our advanced PDAC organoid models were hypoxic and dependent on the USP25-HIF-1α-axis for survival, both in vitro and in vivo, due to the regulation of metabolic rewiring. We found that USP25 depletion drastically altered glycolytic metabolism, characterized by reduced serine, glycine, and lactate biosynthesis. It has previously been reported that cancer cells use up to 50% of their glucose-derived carbon for the production of serine and subsequent folate and nucleotide biosynthesis, while loss of this metabolic shunt leads to cell death specifically in cancer cells^39^. Moreover, KRAS-driven pancreatic models were shown to upregulate enzymes involved in de novo serine synthesis, which was essential for PDAC growth and survival^40^. These observations support our finding that PDAC organoids, and not healthy pancreatic organoids, were sensitive to pharmacological inhibition of USP25.

In PDAC tumors, USP25 is highly expressed and its activity significantly elevated relative to normal quiescent pancreatic tissue, making it an attractive therapeutic candidate. In this study, we used a small molecule inhibitor of USP25 and found that USP25 inhibition dramatically reduced organoid growth and viability at nanomolar concentrations in 13 different PDO lines, suggesting that USP25 may be a promising therapeutic target in PDAC. In support of this, FT206 monotherapy in vivo led to a substantial regression of murine KPCY subcutaneous tumors and PDOXs, with increased tumor cell apoptosis in the hypoxic tumor core. Furthermore, FT206 treatment triggered a marked fall in key glycolytic enzymes and the glucose transporter SLC2A1. Interestingly, neither USP25 nor SLC2A1 were detected in the surrounding stroma compartment, nor did we observe changes in the stroma marker αSMA upon FT206 treatment. Our findings suggest that the USP25-HIF-1α-glycolytic axis is a specific requirement and potential therapeutic vulnerability for PDAC tumor cells. Whether PDAC tumor cells are therapeutically vulnerable to the loss of USP25 in an orthotopic tumor model, which have a greater extent of desmoplasia and angiogenesis, has yet to be explored and warrants further investigation. Of note, FT206, similar to other reported USP25/USP28 inhibitors^41^, is a dual-specificity DUB inhibitor and inhibits both USP25 and USP28 due to their structural homology. However, FT206 mode-of-action in PDAC was dependent on USP25 expression, and significantly reduced organoid viability independently of USP28. Moreover, FT206 treatment phenocopied the effects of *USP25* genetic silencing, including loss of HIF-1α transcriptional signaling, which strongly suggests that FT206 acts on target. Although our data highlights HIF-1α as crucial USP25 substrate in PDAC, it is important to note that USP25 function has been shown to regulate other pathways. Previously, USP25 has been shown to deubiquitinate tankyrase, leading to upregulation of canonical Wnt-β-catenin signaling^36^. However, these pathways were not identified in our RNA-seq as differentially regulated upon *Usp25* silencing, as observed in colorectal cancer^42^. Additionally, targeting these pathways with specific tankyrase and Wnt signaling inhibitors did not mimic the USP25 phenotype in our PDAC organoid models. In summary, our study demonstrates that USP25 is a key mediator of PDAC maintenance and survival through its regulation of HIF-1α protein stability and metabolic rewiring. USP25 represents an exciting therapeutic target and should be considered as a potential therapy for human pancreatic ductal adenocarcinoma.

## Methods

### Human tissue and generation of organoids

Human pancreatic tissues were obtained from King’s College Hospital patients undergoing surgery for pancreatic neoplasia who kindly consented to donate their samples for research. Patients with clinical presentations strongly suggestive of non-exocrine pancreatic tumors were excluded. The study was carried out with formal ethical approval from the Research Ethics Committee (REC, Northern Ireland office) and Health Research Authority (HRA). The study reference ID is IRAS #199628, 16/NI/0119. The study is compliant with all relevant ethical regulations regarding research involving human participants. Human PDAC organoids cultures were established directly from biopsies and cultured as previously described, with variations as describe below^4^. Enrichment for tumor organoids as done by removal of EGF from medium as described previously^5^.

### Maintenance of human pancreatic organoids

Organoids were grown media composed of Advanced DMEM/F12 (Thermo Fisher) supplemented with 100 µ/mL Primocin (InvivoGen), 1x GlutaMAX (Thermo Fisher), 1x B27 supplement (Thermo Fisher), 10 mM Nicotinamide (Sigma-Aldrich), 1.25 mM N-acetylcysteine (Sigma-Aldrich), 10 nM Gastrin (Sigma-Aldrich), 100 ng/mL hFGF-10 (Peprotech), 50 ng/mL EGF (Peprotech), 100 ng/mL mNoggin (Peprotech), 500 nM A83-01 (Tocris biosciences), 10% (v/v) R-Spondin-1 conditioned medium, and 50% (v/v) Afamin-Wnt3a conditioned medium^43^. All conditioned mediums were generated in-house by the Cell Services Laboratory. Human organoids were passaged using TrypLE Express (Gibco) and resuspended in Matrigel (Corning). Organoid medium was supplemented with 10 µM Y-27632, which was removed upon the first medium change (2–3 days).

### Mice

All animal experiments were approved by the Francis Crick Institute Animal Ethics Committee and conformed to UK Home Office regulations under the Animals (Scientific Procedures) Act 1986 including Amendment Regulations 2012. The study is compliant with all relevant ethical regulations regarding animal research. LSL-KRas^G12D^ ^44^, Trp53^Flox/Flox^ ^45^, Pdx1-Cre^46^, Rosa26-LSL-YFP^47^, Pdx1-Flp^48^, FSF-KRas^G12D^ ^48^, Trp53^FRT/FRT^ ^48^, Rosa26-LSL-mTmG^49^, and USP28^flox/flox^^50^ mouse lines have been previously described. These lines were inter-crossed on a mixed background to generate the genotypes of this study. All strains were genotyped by Transnetyx. Adult male immunocompromised NSG and Nu/Nu mice for graft injections have been previously described^51,52^. The mice were housed in constant temperature, humidity, and pathogen-free controlled environment (25 °C ± 2 °C, 50–60%) cage with a standard 12 h light/ 12 h dark cycle, plenty of water, and food (pathogen-free) in their cage. All animal experiments were conducted in adult males, aged 7–12 weeks. For experiments in which mice developed a subcutaneous tumor, the maximum tumor size did not exceed 1.5 cm, in compliance with project license PP9490916.

### Generation and maintenance of primary murine organoids

Mouse primary tumor cells were isolated, grown and passaged in the defined organoid medium as previously described^4^. Briefly, tumors were dissected and placed into ice-cold Advanced DMEM/F-12 containing 50 U ml^−1^ penicillin/streptomycin (Thermo Fisher). Tumors were further minced on ice and incubated with 1 mg ml^−1^ collagenase V (Sigma-Aldrich) solution for 40 min at 37 °C. The solution was filtered through a 70 µm nylon mesh. Following filtration, the organoids were spun down and resuspended in Matrigel. Organoids were passaged every 5–7 days.

### FT206 drug screen in human organoids

FT206 was synthesized and prepared as previously described^34^. Prior to screening, organoids were cultured and dissociated into single cells as described in “Materials and Method” section. Cell number was calculated using the CellTiter-Glo Luminescent Cell Viability Assay (Promega) following the manufacturer’s instructions. Cells were subsequently diluted in 80% human complete medium + 10 µM Y-27632 with 20% Matrigel mixture to a viability reading of approximately 50,000–75,000 units/well (±400 cells). The organoid mixture was plated into ultra low-attachment, flat-bottom 384-well plates (Corning) using a Xrd-384 liquid dispenser (FluidX) at 15 µL/well. Following Matrigel solidification, 75 µL of human complete medium + 10 µM Y-27632 was dispensed per well using the Xrd-384 liquid dispenser. 48h post-seedings, organoids were treated with FT206, AZ6102 (Selleckchem) or DMSO at indicated concentrations using the ECHO 550 acoustic liquid handler (Labcyte). Per organoid line, 24 DMSO vehicle controls were used, and each FT206 dose was done in technical triplicate. Organoids were incubated with drugs for 5 days, then imaged using a high-content screening Cellnsight CX7 (Thermo Fisher). Media was the aspirated using a Biotek ELX405 plate washer, and Cell-Titre Glo reagent was added with the Xrd-384 liquid dispenser. Luminescence was then quantified using the Paradigm plate reader (Molecular Devices). To generate dose-response curves, the average luminescence across DMSO controls was calculated by taking the average of all DMSO values on the plate. A percentage viability across the different drugs and concentrations was then calculated by dividing the luminescence value for each well by the average DMSO control value. The average viability for each drug at each concentration was then calculated by taking the mean viability across the triplicates.

### Generation of arrayed shRNA DUB library and Lentiviral production

The pLKO.1 lentivirus shDUB arrayed library was customed designed and purchased by Merck as arrayed bacterial glycerol stocks. Previously validated shRNA targeting sequences were chosen for each DUB, listed in Supplementary Table 2. Each pLKO.1 plasmid was grown and purified using ZymoPURE II Plasmid Maxiprep kit (Zymo) and correctness verified by sequencing. HEK293T cells were seeded on poly-L-Lysine (Sigma-Aldrich) treated plates. Cells at 70% confluency were co-transfected using JetPRIME (Polyplus) with pLKO.1-shRNA-puro constructs (described in Supplementary Table 2), pMD2.6 G (Gift from Didier Trono, Addgene #12259), and psPAX2 (Gift from Didier Trono, Addgene #12260) at a 1:1:3 DNA concentration ratio. Cells were incubated with transfected mixture in OptiMEM minus antibiotics for 5-6 h. Medium was then replaced with DMEM + GlutaMAX (Gibco) supplemented with 10% FCS (Gibco) and 20 mM HEPES (Gibco). 48 h post transfections, the viral supernatant was collected and filtered twice using 0.45 µm filter (Merck Millipore). Virus titre was calculated using Quicktiter Lentivirus Quantification kit (Cell Biolabs).

### Organoid Lentiviral transduction

Organoids were cultured and dissociated into single cells, passed through a 40 µm filter and resuspended in medium containing 10 µM Y-27632 and Polybrene (8 mg/mL, Sigma-Aldrich). Cells were spin-infected (700*g*, 90 min, 25 °C) on low-adhesion plates (Corning), followed by a rotation incubation at 37 °C for 4–5 h. Following, organoids were centrifuged at 400*g* for 3 min at 4 °C, and seeded in Matrigel. The selection was started 3–5 days post-transduction with either puromycin (2 µg/mL, Gibco) or blasticidin (5 µg/mL, Gibco) where indicated.

### High-content phenotypic screen and caspase-3 assay in organoids

Organoid sphere formation and growth over-time following lentiviral knock-down were achieved using an Incucyte S3 Live-Cell Analysis System (Sartorius). Briefly, 72 h post-viral transduction, organoids were dissociated into single cells, filtered and resuspended into Matrigel as described above. Roughly 100 organoids in 5 µL of Matrigel spots were seeded in black 96-well plate (Greiner Microclear), and 200 µL organoid medium supplemented with 2 µg/mL puromycin was added. For human organoids, cells were seeded directly after viral transduction in a similar manner and recovered for 5 days prior to puromycin selection. Imaging data were collected using a 4x objective, whole-well capture settings in both phase and green-488 fluorescence channels. Percent well confluency quantifications were measured using the built-in analysis on the Incucyte S3 system. Where indicated, detection of caspase-3 activity in live cells was achieved through incubation with the cell-permeable caspase-3 substrate DEVD-NucView488 generated in-house by the Peptide Chemistry Laboratory as described^53^. Both the DEVD-NucView488 caspase-3 and the YFP signal from KPCY organoids were measured and background subtracted using the built-in analysis on the Incucyte S3 system and displayed as mean fluorescent intensity. Following phenotypic imaging, organoid viability was using the CellTiter-Glo Luminescent Cell Viability Assay following manufactures instructions. The values between replicates (*n* = 3) were averaged, and bar graphs display mean ± S.D. Where indicated, media glucose concentration was measured using the FreeStyle Optium Neo (Abbott) glucose meter following manufacturer’s instructions.

### Tumor volume measurement using 3D ultrasound

3D ultrasound images of tumors were acquired using Microscan^TM^ transducer MS-550D and a 3D stage control system (VEVO 2100; Visualsonics). Under anesthesia (1.5–2.5% isoflurane in oxygen 1.5–2 L/min), mice were placed in a lateral position on the heated (37 °C) ultrasound platform. The hair from the flanks area was removed by hair removal cream (Veet). Continuous 2D-mode images were acquired by scanning across the subcutaneous tumor at intervals of 0.2 mm, followed by 3D volume reconstruction. The whole procedure took approximately 10 min. The body temperature, heart rate and respiration rate were monitored throughout image acquisition. 3D tumor volume was analyzed using the 3D analysis tool, Vevo lab software version 3.2.0.

### In vivo pharmacology with subcutaneous and xenograft tumors

For in vivo murine studies FT206 was prepared to a concentration of 75 mg/kg in 10% (v/v) ethanol and 90% (v/v) PEG300 (Sigma-Aldrich), as previously described^34^. Mouse (KPCY-derived) and Human organoid lines (PDOs) were resuspended as single-cell suspensions at 1^7^ cells/ml in Matrigel. 100 μl (1^6^ cells total) of this suspension was injected into the flanks of immunodeficient Nu/Nu or NSG mice. When tumors were palpable (>50–100 mm^3^), mice were treated with FT206 (75 mg/kg) via oral gavage, three times per week for 5 weeks. Body weights were registered three times a week. Tumor volumes were determined using 3D ultrasound and measurements were means ± standard deviation.

### RNA isolation and quantitative PCR (RT-qPCR)

RNA was extracted using the RNeasy Mini Kit (Qiagen) according to the manufacturer’s instructions. One microgram of total RNA was reverse-transcribed using the iScript cDNA synthesis kit (BioRad) according to the manufacturer’s instructions. cDNA was diluted tenfold and 4 μL was used per RT-qPCR reaction. PowerUp SYBRGreen (Applied Biosystems) was used for real-time quantitative PCR (qPCR). Assays were performed on a Via7 (Applied Biosystems). Primers were designed using the Universal Probe Library Assay Design Center (Roche), ensuring that they span exon–exon junctions. Gene expression levels were determined and normalized to the geomean expression levels of two reference genes, 36B4/36b4 and TBP/Tbp. RT-qPCR primers are listed in Supplementary Table 3.

### RNA sequencing-based transcriptional profiling

KPCY organoids were transduced with lentiviruses targeting YFP or Usp25, recovered and placed under puromycin selection as described above. Roughly, 30 h post-puromycin selection organoids were dissociated, lysed and total RNA was isolated using method described above. RNA was converted into strand-specific cDNA libraries using the KAPA mRNA HyperPrep kit (Roche) according to the manufacturer’s instructions. The libraries were analyzed on an Illumina HiSeq 4000 and subsequently sequenced with single ended 75 bp reads with around 25–30 million reads per sample. Adaptor trimming was performed with Trimmomatic/0.36-Java-1.7.0_80 with parameters “LEADING:3 TRAILING:3 SLIDINGWINDOW:4:20 MINLEN:36”. The RSEM package (v.1.2.31)^54^ in conjunction with the STAR alignment algorithm (v.2.5.2a)^55^ was used for the read mapping and gene-level quantification with respect to mouse Ensembl GRCm38 - release 89. The parameters used were: –star-output-genome-bam –forward-prob 0. Differential expression analysis was performed with the DESeq2 package (v.1.20.0)^56^ within the R programming environment (https://www.r-project.org/; v.3.5.1), where the significance threshold for the identification of differentially expressed transcripts was set to an adjusted *P* value ≤0.05. Principal component analysis (PCA) was performed on log-transformed normalized counts for the top 5000 most variable transcripts (as determined by standard deviation) using R with the pcaMethods package (v.1.82.0)^57^. Hierarchical clustering was performed on log-transformed and mean-centered normalized counts for all differentially expressed transcripts using Cluster 3.0^58^. Pathway analysis was performed on all differentially expressed, protein-coding transcripts with a Log_2_FC < −0.5 using Enrichr^59^. The top pathways are shown from the HALLMARK and KEGG (Kyoto Encyclopedia of Genes and Genomes) databases. Heatmaps of gene expression z-scores were generated for the KEGG pathways Glycolysis/Gluconeogenesis (KEGG: hsa00010) and HIF-1 signaling (KEGG: hsa04066), and for the HALLMARK pathway Hypoxia (ID:M5891). The RNA-seq dataset is available on the GEO database, GSE166077.

### CRISPR/Cas9-mediated knock-out of USP25 in patient-derived organoids

The CRISPR/Cas9 system was utilized to delete USP25 in patient-derived organoid (PDO) lines K3T, K4T, K13T, and K17T. The protocol for engineering PDOs by electroporation was adapted and modified from^60^. Organoids were dissociated to single cells as described above and diluted to 1×10^6^ cells/electroporation. The cell pellet was then resuspended to precisely 100 µL of solution composed of OptiMEM with 7.2 µg PB-CMV-MCS-EF1a-RFP-T2A-Puro (System Biosciences), 2.8 µg CMV-pHyPBase^61^, and 10 µg PX330 (Gift from Feng Zhang, Addgene #42230^62^). Cell/DNA mixtures were transferred into 2 mm electroporation cuvettes (Nepagene) then electroporated using a NEPA21 super electroporator (Nepagene), using the settings described in Supplementary Table 4. The electroporated cell suspension was then transferred to human complete medium supplemented with 10 µM Y-27632. Organoids were then centrifuged, resuspended in Matrigel, and grown in human complete medium supplemented with 10 µM Y-27632 and 3 µM CHIR-99021 (Selleckchem). Media was subsequently changed every 2–3 days. CHIR99021 was removed day 5 post-electroporation. On day 7 post-electroporation, organoids were selected with 2 µg/mL puromycin. 5–7 days post-selection, organoids were either assessed for viability using Cell-Titre Glo assay, or expanded for single clone knock-out lines. To generate clonal organoid lines, electroporated organoids were dissociated to single cells and plated at low density. After recovery and expansion, individual RFP + organoids were handpicked under a microscope. Organoids were then expanded until enough material was available to extract gDNA. gDNA was extracted using the Purelink Genomic DNA Minikit per the manufacturer’s instructions (Thermo Fisher Scientific). The USP25 exon of interest was then sequenced with region-specific primers and knock-out was verified by Sanger sequencing, as well as by immunoblotting.

### Generation of Usp28 knock-out PDAC organoids

Organoids were generated as described above from tumor-bearing mice containing the following alleles: Pdx1-Flp, FSF-KRas^G12D^, Trp53^FRT/FRT^, Rosa26-LSL-mTmG, and USP28^flox/flox^. Organoids were transduced with adenoviruses encoding Cre recombinase (Adeno-CMV-Cre; UI viral vector core, VVC-U of Iow-5-HT) at an MOI of 1:50. Single knock-out clones were sorted based on GFP expression, driven by the Rosa26-LSL-mTmG reporter, and knock-out was validated by immunoblot.

### Cell Culture and siRNA interference experiments

Human HEK293T, PANC-1, AsPC-1 and RCC-4 cell lines were obtained from the Francis Crick Institute Cell Services, and certified negative for mycoplasma. Cells were maintained in Dulbecco’s modified Eagle’s medium (DMEM) supplemented with 10% FBS, and 1% Penicillin/Streptomycin at 37 °C with 5% CO_2_. Generation of RCC-4 cell lines expressing the VHL gene, or EV control, were described in^63^. To silence human *USP25*, ON-TARGETplus individual USP25 siRNAs (Horizon Discovery, LQ-006074-00-0005) and a non-targeting control (Horizon Discovery, D-001810-01-05) were used. Cells were reversed-transfected with 30 nM small interfering RNAs (siRNAs) using Lipofectamine RNAiMAX (Invitrogen) for 48–72h. Where indicated, 48 h post-siRNA transfections cells were treated with 25 µM MG132 (Sigma-Aldrich) for 8 h prior to analysis.

### Immunohistochemistry

Tissues were collected, fixed in 10% neutral-buffered formalin (NBF, Sigma-Aldrich) for 16 h, dehydrated in 70% ethanol and embedded in 4 µm paraffin sections. The slides were de-paraffinized in xylene and rehydrated using a series of graded industrial methylated spirits solutions to distilled water. No antigen retrieval was performed for USP25 staining. Heat-mediated antigen retrieval was done in 10 mM sodium citrate buffer (pH 6.2) for SLC2A1 staining. Endogenous peroxidase blocking was performed using 1.6% H2O2 for 10 minutes at room temperature (RT) and protein blocking was performed using 2.5% Normal Horse Serum (ready-to-use; MP-7401, Vector Laboratories) overnight at 4 C. Primary antibodies was diluted in 1% BSA, and incubated overnight at 4 °C; after washing in PBS, slides were incubated in HRP Horse Anti-Rabbit IgG Polymer (MP-7401, Vector Laboratories) for 30 min at RT. Finally, the slides were developed in 3,3-diaminobenzidine (DAB) chromogen (SK-4100, Vector Laboratories) for 10 minutes at RT. The slides were counterstained with Harris Heamatoxilin (3801561E, Leica Biosystems), dehydrated, cleared, and mounted in a Sakura Tissue-Tek Prisma^®^ auto stainer. The Caspase-3, CK19, and GFP IHC was performed on the Discovery Ultra Ventana platform (from Roche). All primary antibodies and dilutions are described in Supplementary Table 5.

### Immunofluorescence staining

Tissues were collected and fixed, embedded and sectioned as described above. Slides were dewaxed with Histo-Clear (National Diagnostic) and dehydrated using a series of graded industrial ethanol solutions to distilled water. Heat-mediated antigen retrieval was done in 10 mM sodium citrate buffer (pH 6.2) and blocking of endogenous peroxidase was achieved with 1% BSA in PBS + 5% FCS. Tissues were incubated overnight with primary antibodies, described in Supplementary Table 5, as well as 0.3 µM DAPI (Sigma-Aldrich). Following, slides were washed 3× with PBS and incubated with secondary antibodies for 1 h at room temperature. Where indicated, slides were treated with 10 µg/mL Lycopersicon Esculentum (Tomato) Lectin-DyLight-488 (Vector Laboratories, DL-1174), following manufactures instructions. For hypoxia staining, animals were dosed with 60 mg/kg body weight hypoxyprobe reagent (Hypoxyprobe Inc.), following manufactures instructions. Tissues were embedded with O.C.T. compound (Tissue-Tek), and 5 µm frozen sections were stained overnight with mouse IgG1 monoclonal antibody clone 4.3.11.3 (Hypoxyprobe Inc). Background fluorescence was blocked with Sudan black incubation, and slides were mounted with fluorescent mounting medium (Dako). Tissue slides were processed using the Zeiss Axio Scan.Z1 slide scanner with Zen 3.0 software package. Fluorescence intensity quantifications were measured using ImageJ software.

### Over-expression plasmids and DNA transfections in cell lines

Expression plasmid encoding human USP25 cDNAs were generated with Gateway-mediated recombination (Invitrogen). The activity disabling C187S mutation in USP25 was introduced using QuickChange site-directed mutagenesis kit (Agilent). V5/His‐tagged expression plasmids encoding human HIF-1α were kindly provided by Ya-Min Tian (University of Oxford), and HA-tagged human HIF-1α was a gift from William Kaelin (Addgene #18949)^64^. All plasmids used in this study were isolated using ZymoPURE II Plasmid Maxiprep kit (Zymo) and correctness verified by sequencing. Hek293T cells were seeded for 70% confluency at the time of transfection on poly-L-Lysine treated plates. Indicated plasmids were transfected using Jetprime, and a GFP expressing plasmid was used a transfection efficiency control (over 85-95% efficiency). Where indicated, cells were treated with 200 µM CoCl_2_ (Sigma-Aldrich) for 24 h. Samples were collected and processed 48 h post transfections. For sub-cellular fractionation, cells were processed using the NE-PER nuclear and cytoplasmic extraction reagent kit (Thermo Fisher Scientific), per the manufacturer’s instructions.

### Generation of doxycycline-inducible over-expressing patient-derived organoids

Gateway-mediated recombination was used to clone USP25 wild-type, USP25^C187S^, or HIF1A^P402A/P456A^ (Gift from Eric Huang, Addgene #52636^65^) into the lentivirus pInducer20-Blast vector (Gift from Jean Cook, Addgene #109334^66^). Lentivirus vectors were amplified, purified, sequenced and lentivirus containing particles were generated as described above. Single dissociated human organoids were transduced as described above, and clonally selected with 5 µg/mL blasticidin. To induce *USP25* over-expression, organoids were treated with 1 µg/mL doxycycline for a period of 48–72 h.

### Immunoblot analysis

Total cell lysates were prepared in radioimmunoprecipitation assay (RIPA) buffer (Cell Signaling) supplemented with protease inhibitors (Sigma-Aldrich), phenylmethylsulfonyl fluoride (Sigma-Aldrich) and PhosSTOP (Roche). Lysates were cleared by centrifugation at 4 °C for 10 min at 10,000*g*. Protein concentration was determined using a Detergent-compatible colorimetric assay kit (Biorad). Samples (10-40 µg) were separated on mini-PROTEAN TGX 7.5% gels (Biorad) and transferred to nitrocellulose membranes. Non-specific binding was blocked using 5% nonfat dry milk in TBS-Tween (0.2%) for 1 hr at room temperature. The membranes were incubated with the primary antibodies listed in Supplementary Table 5. Following 3 × 5 min TBS-Tween washes, membranes were blotted with secondary antibodies conjugated with horseradish peroxidase for 1 hr (1:10,000; Sigma-Aldrich). The washed membranes were incubated with either ECL Western Blotting Detection reagent (Amersham) or SuperSignal West Femto Chemiluminescent substrate (ThermoFisher), and visualized with chemiluminescence on a Fuji LAS4000 (GE Healthcare). Where indicated, fold-change was measured using ImageJ software and normalized by loading controls from the same experiments that were processed in parallel.

### Co-immunoprecipitation assays

Cells were lysed in Cell Lysis Buffer (Cell Signaling) supplemented with protease inhibitors, phosphatase inhibitors. Samples were incubated on ice for 30 min, sonicated briefly for 5 seconds, and debris was precleared by centrifugation (4 °C for 10 min at 10,000*g*). Protein concentrations were measured as described above and concentrations were adjusted to 1 mg/mL. For USP25 immunoprecipitation, samples incubated overnight (rotating at 4 °C) with 4 µg of USP25 antibody (Abcam, ab187156) or 4 µg rabbit IgG control (Abcam, ab172730). Samples then rotated 5 h at 4 °C with 30 µL of sheep-anti-rabbit magnetic beads (Merck). For HA-tagged immunoprecipitation, samples incubated overnight (rotating at 4 °C) with 30 µL EZview Red Anti-HA affinity agarose (Merck). For all, samples were washed 4× in wash buffer (50 mM Tris pH 7.5, 150 mM NaCl_2_, 1 mM EDTA, 1% Triton X-100, 5% glycerol), and eluted by boiling in 60 µL 2× SDS loading dye.

### Agarose-TUBEs assays

Cells were lysed in lysis buffer (50 mM Tris-HCl pH 7.5, 150 mM NaCl, 1 mM EDTA, 1% NP-40, 10% glycerol) supplemented with protease inhibitors, 1 mM DTT, 50 µM PR-619 (LifeSensors), 50 mM 1,10-phenanthroline (LifeSensors). Samples were incubated on ice for 30 min, and debris was precleared by centrifugation (4 °C for 10 min at 10,000*g*). Protein concentrations were measured as described above, and concentrations were adjusted to 1 mg/mL and incubated with 20 µL of Agarose-TUBE slurry (LifeSensors). Immunoprecipitations were performed overnight, rotating at 4 °C. Following, resin was washed 4x in wash buffer (50 mM Tris pH 7.5, 150 mM NaCl, 0.1% Triton X-100, and 5% glycerol), and bound proteins were eluted by boiling in 50 µL 2x SDS loading dye.

### Cy5-UB-PA/Cy5-UB-VME DUB activity probe labeling

The Cy5-UB-PA (UbiQ, UbiQ-072) and Cy5-UB-VME (UbiQ, UbiQ-071) probes were prepared based on manufactures instructions. KPCY organoids and PDOs were harvested as described above, and lysed in lysis buffer (50 nM Tris, 250 mM sucrose, 5 mM MgCl_2_, 1 mM DTT, 0.5% CHAPS, 0.1% Nonidet P-40). Samples were incubated on ice for 30 min, and debris was precleared by centrifugation. Protein concentrations were measured using the Detergent-Compatible Colorimetric Assay Kit, and a total of 25 µg of lysate was incubated with 200 nM PR-619 or DMSO vehicle control for 15 min at room temperature. Subsequently, 1 µM of Cy5-UB-PA and Cy5-UB-VME and 50 nM NaOH were added to the samples and incubated for 15 min in the dark at room temperature. Sample buffer was added, samples were boiled and loaded onto SDS-PAGE gels as described above. Fluorescence Cy5 signal was imaged in-gel using Fuji LAS4000.

### DUB activity-based probe and mass spectrometry

Organoids were cultured and dissociated as described above. Cells were homogenized in cold DUB activity buffer (50 mM TRIS [pH7.5], 150 mM NaCl, 0.5% CHAPS, 0.1% NP40, 5 mM MgCl_2_, 10% Glycerol, 1 mM DTT,). Mouse KPCY tumors or normal pancreatic tissues were collected from 7 to 9 week old adult mice. Tissues were homogenized using a MagNA lyser (Roche) in cold DUB activity buffer following manufactures instructions. All supernatants were collected following centrifugation (4 °C for 15 min at 10,000*g*). Protein concentrations were measured as described above and adjusted to 1 mg/mL. Samples were treated with PR619 or DMSO vehicle control, at a final concentration of 50 µM. All samples were incubated for 1 h at 25 °C with 2 µM equal mixture of DUB activity-based probes Biotin-ubiquitin-propargylamide (Biotin-Ahx-UB-PA, UbiQ, UbiQ-076), Biotin-ubiquitin-vinyl methyl ester (Biotin-Ahx-UB-VME, UBiQ, UbiQ-054), and Biotin-ubiquitin-vinylsulfone (Biotin-Ahx-UB-VS, UbiQ, UbiQ-188). The excess probe was then removed with 10 K centrifugal column filtration (Merck Millipore), and samples were diluted in IP buffer (0.2% SDS/PBS) to a volume of 1 mL/condition and incubated with 100 µL High Capacity Neutravidin Agarose resin (Thermo Fisher Scientific) modified in-house by dimethylation of lysine residues prior to use. The samples were rotated at room temperature for 3 h, and then washed 2× with DUB activity buffer, 2× with 4 M Urea/PBS, and 3× with PBS. For the label-free experiments in organoids, proteins were eluted from the beads with 50 µL 2× LSB, heated at 95 °C for 5 min and run on 10% SDS-PAGE gels. Following a short-run (1 cm protein front) gels were stained with InstantBlue (Sigma-Aldrich) and entire lanes alkylated prior to lys-C digestion overnight in 50 mM TriEthylAmmonium Bicarbonate (TEAB) pH 8. Supernatants were removed and digested with trypsin for 3 h (ThermoFisher Scientific). Proteolysed samples were vacuum centrifuged and resuspended in 0.1% TriFluoroAcetic acid (TFA). This approach prevents the excessive levels of digested neutravidin observed with unmodified beads. For the TMT experiments in primary murine tissues, samples were alkylated then digested on-bead with Lys-c and trypsin, as above, in a thermomixer at 800 rpm. TMT 10plex (ThermoFisher Scientific) isotopic labeling was performed according to the manufacturer’s instructions. Samples were TMT labeled as follows: 126, 127N, 127C = murine WT pancreatic tissue replicates; 128N, 128C, 129N = murine PDAC tumor replicates; 129C, 130N, 130C = murine PDAC tumor + DUBi replicates; 131 = unused. After checking labeling efficiency was >90%, differentially labeled samples were combined and desalted with 3 M Empore^TM^ C18 solid phase extraction discs (aka “stage tips”) prior to drying and resuspension as with the label-free experiments. On an Ultimate 3000 nanoRSLC HPLC resuspended peptides were loaded on a 2 mm × 0.3 mm Acclaim Pepmap C18 trap column prior to the trap being switched to elute at 0.25 µl/min through a 50 cm × 75 um EasySpray C18 column into an Orbitrap Fusion Lumos Tribrid Mass Spectrometer (HPLC, columns and MS all from ThermoFisher Scientific). Peptides were eluted with a gradient of 2–40%B in 60′ (LFQ) or 150′ (TMT), followed by a cleaning step with 100%B and re-equilibration (A = 2%ACN,0.1% formic acid; B = 80%ACN, 0.1% formic acid). The orbitrap was operated in “Data Dependent Acquisition” mode with a survey scan at a resolution of 120k from *m*/*z* 300–1500, followed by MS/MS in “TopS” mode. Dynamic exclusion was used with a time window of 20 s. The Orbitrap charge capacity was set to a maximum of 1e6 ions in 10 ms. HCD was adopted for MS/MS fragmentation. The label-free samples were acquired in the ion trap (1e4 ions in 100 ms). TMT labeled samples were acquired in the orbitrap at 50k resolution (1e5 ions in 100 ms). Raw files were processed using Maxquant (v2.0.3.1) and Perseus (v2.0.3.0) and recent downloads of the human or mouse Uniprot reference proteomes. A decoy database of reversed sequences was used to filter false positives, at a peptide false detection rate of 1%. The mass spectrometry proteomics data have been deposited to the ProteomeXchange Consortium via the PRIDE^67^ partner repository with the dataset identifier PXD023424.

### Metabolite extraction and gas chromatography-mass spectrometry

After shYFP control or shUsp25 infection, KPCY organoids recovered for 72h, and were selected with 2 µg/mL puromycin for 30h, as described above. Media was then swapped for organoid medium containing 4 mM U-^13^C -glucose (Cambridge Isotype Laboratories) for 6 h. Metabolites were extracted using methanol/chloroform mixture with 1 mmol *scyllo*-inositol (Sigma) as an internal standard. Cell debris was used to extract and quantify total DNA content using the PureLink Genomic DNA Mini Kit (Thermo Fisher) according to manufacturer’s instructions. Metabolites were partitioned by resuspension in 350 µL chloroform/methanol/water (1:3:3, v/v), and the upper phase (containing polar metabolites) was dried in a GC-MS vial insert (Agilent). The dried extract was then resuspended in MeOH and subsequently dried. This step was repeated another time. Data acquisition was performed largely as previously described^68^, using an Agilent 7890B-7000C GC-MSD in EI mode after derivatization of dried extracts by addition of 20 µL methoxyamine hydrochloride (20 mg/mL in pyridine, Sigma, RT, > 16 hr) and 20 µL BSTFA + 1% TMCS (Sigma, RT, > 1 hr. GC-MS parameters were as follows: carrier gas, helium; flow rate 0.9 mL/min; column, DB-5MS (Agilent); inlet, 270 °C; temperature gradient, 70 °C (2 min), ramp to 295 °C (12.5 °C/min), ramp to 320 °C (25 °C/min, 3 min hold). Scan range was *m*/*z* 50-550. Data analysis was performed using MANIC software version 1.0^69^. Metabolites were identified and quantified by comparison to authentic standards, and label incorporation estimated as the percentage of the metabolite pool containing one or more 13C atoms after correction for natural abundance. Total metabolite abundance was normalized to the corresponding DNA content of the replicate.

### Meta-analysis of expression and survival datasets

Transcript expression of *USP25* in PDAC tumors and adjacent normal tissues, and Spearman correlations were evaluated and computed using GEPIA (Gene Expression Profiling Interactive Analysis; http://gepia.cancer-pku.cn/)^70^ of TCGA (The Cancer Genome Atlas and GTEx (Genotype Tissue Expression) datasets. Patient prognoses were evaluated by Kaplan-Meier survival curves of Pancreatic ductal adenocarcinoma patients with low or high expression of USP25 using the Kaplan-Meier plotter online platform (http://kmplot.com)^71^. The database includes RNA-seq information based on TCGA and GEO datasets.

### Statistics and reproducibility

Results are represented as mean ± standard deviation (SD) for all bar graphs and median plus interquartile range for violin plots. Statistical significance was determined as described in the figure legends and calculated using GraphPad Prims 9. Briefly, normal distribution of data was tested using the Shapiro–Wilk normality test. *P*-values were calculated using unpaired, two-tailed Student’s *t* test followed by correction for multiple comparison using Holm-Sidak post-hoc test where applicable. Alternatively, one- or two-way analysis of variance followed by Dunnett’s or Tukey’s correction for multiple comparisons was used. *P*-values are provided in the figure panels or, if not possible, in the figure legends. A *p*-value of <0.05 was regarded as statistically significant for all datasets. The exact sample sizes (*n*) used to calculate statistics are provided in the figure legends. All experiments were reproduced with similar results a minimum of three times. All immunoblots and micrograph images are representative of a minimum of three biologically independent experiments or samples.

### Reporting summary

Further information on research design is available in the Nature Research Reporting Summary linked to this article.

## Supplementary information

Supplementary Information

Reporting Summary

## Data Availability

The RNA sequencing data that supported the findings of this study are available on the GEO database, GSE166077. The mass spectrometry proteomics data have been deposited to the ProteomeXchange Consortium via the PRIDE partner repository with the dataset identifier PXD023424. The remaining data are available within the Article, Supplementary Information or Source Data file. Source data are provided with this paper.

## Source data

Source Data

## References

[1] Siegel RL, Miller KD, Jemal A (2019). Cancer statistics, 2019. CA Cancer J. Clin..

[2] Cronin KA (2018). Annual report to the nation on the status of cancer, part I: National cancer statistics. Cancer.

[3] Hezel AF, Kimmelman AC, Stanger BZ, Bardeesy N, Depinho RA (2006). Genetics and biology of pancreatic ductal adenocarcinoma. Genes Dev..

[4] Boj SF (2015). Organoid models of human and mouse ductal pancreatic cancer. Cell.

[5] Seino T (2018). Human pancreatic tumor organoids reveal loss of stem cell niche factor dependence during disease progression. Cell Stem Cell.

[6] Tiriac H (2018). Organoid profiling identifies common responders to chemotherapy in pancreatic cancer. Cancer Discov..

[7] Driehuis, E. et al. Pancreatic cancer organoids recapitulate disease and allow personalized drug screening. *Proc. Natl Acad. Sci. USA*, 10.1073/pnas.1911273116 (2019). 10.1073/pnas.1911273116 PMC6936689 31818951

[8] Huang L (2015). Ductal pancreatic cancer modeling and drug screening using human pluripotent stem cell– and patient-derived tumor organoids. Nat. Med..

[9] Barglow KT, Cravatt BF (2007). Activity-based protein profiling for the functional annotation of enzymes. Nat. Methods.

[10] de Jong A (2012). Ubiquitin-based probes prepared by total synthesis to profile the activity of deubiquitinating enzymes. Chembiochem.

[11] Ekkebus R (2013). On terminal alkynes that can react with active-site cysteine nucleophiles in proteases. J. Am. Chem. Soc..

[12] Hewings DS, Flygare JA, Bogyo M, Wertz IE (2017). Activity-based probes for the ubiquitin conjugation-deconjugation machinery: new chemistries, new tools, and new insights. FEBS J..

[13] Clague MJ, Urbé S, Komander D (2019). Breaking the chains: deubiquitylating enzyme specificity begets function. Nat. Rev. Mol. Cell Biol..

[14] Komander D, Clague MJ, Urbe S (2009). Breaking the chains: structure and function of the deubiquitinases. Nat. Rev. Mol. Cell Biol..

[15] D’Arcy P, Wang X, Linder S (2015). Deubiquitinase inhibition as a cancer therapeutic strategy. Pharmacol. Therapeutics.

[16] D’Arcy P, Linder S (2014). Molecular pathways: translational potential of deubiquitinases as drug targets. Clin. Cancer Res..

[17] Harrigan JA, Jacq X, Martin NM, Jackson SP (2018). Deubiquitylating enzymes and drug discovery: emerging opportunities. Nat. Rev. Drug Discov..

[18] Borodovsky A (2002). Chemistry-based functional proteomics reveals novel members of the deubiquitinating enzyme family. Chem. Biol..

[19] Altun M (2011). Activity-based chemical proteomics accelerates inhibitor development for deubiquitylating enzymes. Chem. Biol..

[20] Yan, L., Raj, P., Yao, W. & Ying, H. Glucose metabolism in pancreatic cancer. *Cancers (Basel)* **11**, 10.3390/cancers11101460 (2019). 10.3390/cancers11101460 PMC6826406 31569510

[21] Koong AC (2000). Pancreatic tumors show high levels of hypoxia. Int. J. Radiat. Oncol. Biol. Phys..

[22] Zhong H (1999). Overexpression of hypoxia-inducible factor 1alpha in common human cancers and their metastases. Cancer Res..

[23] Zhu H (2014). Upregulation of autophagy by hypoxia-inducible factor-1alpha promotes EMT and metastatic ability of CD133+ pancreatic cancer stem-like cells during intermittent hypoxia. Oncol. Rep..

[24] Guillaumond F (2013). Strengthened glycolysis under hypoxia supports tumor symbiosis and hexosamine biosynthesis in pancreatic adenocarcinoma. Proc. Natl Acad. Sci. USA.

[25] Kurahara H (2018). Significance of glucose transporter type 1 (GLUT-1) expression in the therapeutic strategy for pancreatic ductal adenocarcinoma. Ann. Surg. Oncol..

[26] Shukla SK (2017). MUC1 and HIF-1alpha signaling crosstalk induces anabolic glucose metabolism to impart gemcitabine resistance to pancreatic cancer. Cancer Cell.

[27] Ben Q (2020). A nicotine-induced positive feedback loop between HIF1A and YAP1 contributes to epithelial-to-mesenchymal transition in pancreatic ductal adenocarcinoma. J. Exp. Clin. Cancer Res..

[28] Li Y, Sun XX, Qian DZ, Dai MS (2020). Molecular crosstalk between MYC and HIF in cancer. Front. Cell Dev. Biol..

[29] Huang LE (2008). Carrot and stick: HIF-alpha engages c-Myc in hypoxic adaptation. Cell Death Differ..

[30] Dang CV, Kim JW, Gao P, Yustein J (2008). The interplay between MYC and HIF in cancer. Nat. Rev. Cancer.

[31] Gordan JD, Thompson CB, Simon MC (2007). HIF and c-Myc: sibling rivals for control of cancer cell metabolism and proliferation. Cancer Cell.

[32] Mole DR, Pugh CW, Ratcliffe PJ, Maxwell PH (2002). Regulation of the HIF pathway: enzymatic hydroxylation of a conserved prolyl residue in hypoxia-inducible factor alpha subunits governs capture by the pVHL E3 ubiquitin ligase complex. Adv. Enzym. Regul..

[33] Maxwell PH, Pugh CW, Ratcliffe PJ (2001). The pVHL-hIF-1 system. A key mediator of oxygen homeostasis. Adv. Exp. Med. Biol..

[34] Ruiz, E. J. et al. USP28 deletion and small-molecule inhibition destabilizes c-MYC and elicits regression of squamous cell lung carcinoma. *Elife* **10**, 10.7554/eLife.71596 (2021). 10.7554/eLife.71596 PMC8553340 34636321

[35] Sauer F (2019). Differential oligomerization of the deubiquitinases USP25 and USP28 regulates their activities. Mol. Cell.

[36] Xu D (2017). USP25 regulates Wnt signaling by controlling the stability of tankyrases. Genes Dev..

[37] Tiwari A (2020). Loss of HIF1A from pancreatic cancer cells increases expression of PPP1R1B and degradation of p53 to promote invasion and metastasis. Gastroenterology.

[38] Lee KE (2016). Hif1a deletion reveals pro-neoplastic function of B cells in pancreatic neoplasia. Cancer Discov..

[39] Locasale JW (2011). Phosphoglycerate dehydrogenase diverts glycolytic flux and contributes to oncogenesis. Nat. Genet..

[40] Maddocks ODK (2017). Modulating the therapeutic response of tumours to dietary serine and glycine starvation. Nature.

[41] Wrigley JD (2017). Identification and characterization of dual inhibitors of the USP25/28 deubiquitinating enzyme subfamily. ACS Chem. Biol..

[42] Wang X-M (2020). The deubiquitinase USP25 supports colonic inflammation and bacterial infection and promotes colorectal cancer. Nat. Cancer.

[43] Mihara, E. et al. Active and water-soluble form of lipidated Wnt protein is maintained by a serum glycoprotein afamin/alpha-albumin. *Elife* **5**, 10.7554/eLife.11621 (2016). 10.7554/eLife.11621 PMC4775226 26902720

[44] Jackson EL (2001). Analysis of lung tumor initiation and progression using conditional expression of oncogenic K-ras. Genes Dev..

[45] Marino S, Vooijs M, van Der Gulden H, Jonkers J, Berns A (2000). Induction of medulloblastomas in p53-null mutant mice by somatic inactivation of Rb in the external granular layer cells of the cerebellum. Genes Dev..

[46] Hingorani SR (2003). Preinvasive and invasive ductal pancreatic cancer and its early detection in the mouse. Cancer Cell.

[47] Srinivas S (2001). Cre reporter strains produced by targeted insertion of EYFP and ECFP into the ROSA26 locus. BMC Dev. Biol..

[48] Schonhuber N (2014). A next-generation dual-recombinase system for time- and host-specific targeting of pancreatic cancer. Nat. Med..

[49] Muzumdar MD, Tasic B, Miyamichi K, Li L, Luo L (2007). A global double-fluorescent Cre reporter mouse. Genesis.

[50] Diefenbacher ME (2014). The deubiquitinase USP28 controls intestinal homeostasis and promotes colorectal cancer. J. Clin. Invest..

[51] Flanagan SP (1966). ‘Nude’, a new hairless gene with pleiotropic effects in the mouse. Genet Res..

[52] Shultz LD (2005). Human lymphoid and myeloid cell development in NOD/LtSz-scid IL2R gamma null mice engrafted with mobilized human hemopoietic stem cells. J. Immunol..

[53] Cen H, Mao F, Aronchik I, Fuentes RJ, Firestone GL (2008). DEVD-NucView488: a novel class of enzyme substrates for real-time detection of caspase-3 activity in live cells. FASEB J..

[54] Li B, Dewey CN (2011). RSEM: accurate transcript quantification from RNA-Seq data with or without a reference genome. BMC Bioinform..

[55] Dobin, A. & Gingeras, T. R. Mapping RNA-seq Reads with STAR. *Curr. Protoc. Bioinformatics* **51**, 11 14 11–19, 10.1002/0471250953.bi1114s51 (2015). 10.1002/0471250953.bi1114s51 PMC4631051 26334920

[56] Love MI, Huber W, Anders S (2014). Moderated estimation of fold change and dispersion for RNA-seq data with DESeq2. Genome Biol..

[57] Thevenot EA, Roux A, Xu Y, Ezan E, Junot C (2015). Analysis of the human adult urinary metabolome variations with age, body mass index, and gender by implementing a comprehensive workflow for univariate and OPLS statistical analyses. J. Proteome Res..

[58] de Hoon MJ, Imoto S, Nolan J, Miyano S (2004). Open source clustering software. Bioinformatics.

[59] Chen EY (2013). Enrichr: interactive and collaborative HTML5 gene list enrichment analysis tool. BMC Bioinform..

[60] Fujii M, Matano M, Nanki K, Sato T (2015). Efficient genetic engineering of human intestinal organoids using electroporation. Nat. Protoc..

[61] Yusa K, Zhou L, Li MA, Bradley A, Craig NL (2011). A hyperactive piggyBac transposase for mammalian applications. Proc. Natl Acad. Sci. USA.

[62] Cong L (2013). Multiplex genome engineering using CRISPR/Cas systems. Science.

[63] Sugimoto, Y. & Ratcliffe, P. J., 10.1101/2021.07.06.451052 (2021).

[64] Kondo K, Klco J, Nakamura E, Lechpammer M, Kaelin WG (2002). Inhibition of HIF is necessary for tumor suppression by the von Hippel-Lindau protein. Cancer Cell.

[65] Kageyama Y (2004). Leu-574 of human HIF-1alpha is a molecular determinant of prolyl hydroxylation. FASEB J..

[66] Matson, J. P. et al. Rapid DNA replication origin licensing protects stem cell pluripotency. *Elife* **6**, 10.7554/eLife.30473 (2017). 10.7554/eLife.30473 PMC5720591 29148972

[67] Perez-Riverol Y (2019). The PRIDE database and related tools and resources in 2019: improving support for quantification data. Nucleic Acids Res..

[68] MacRae JI (2013). Mitochondrial metabolism of sexual and asexual blood stages of the malaria parasite Plasmodium falciparum. BMC Biol..

[69] Behrends V, Tredwell GD, Bundy JG (2011). A software complement to AMDIS for processing GC-MS metabolomic data. Anal. Biochem..

[70] Tang Z (2017). GEPIA: a web server for cancer and normal gene expression profiling and interactive analyses. Nucleic Acids Res..

[71] Nagy A, Lanczky A, Menyhart O, Gyorffy B (2018). Validation of miRNA prognostic power in hepatocellular carcinoma using expression data of independent datasets. Sci. Rep..

