# Supplementary material for: USP25 promotes pathological HIF-1-driven metabolic reprogramming and is a potential therapeutic target in pancreatic cancer
Source: Nat Commun. 2022 Apr 19;13:2070. doi: 10.1038/s41467-022-29684-9 (PMC9018856; doi:10.1038/s41467-022-29684-9)

**SUPPLEMENTARY INFORMATION**

# USP25 promotes pathological HIF-1-driven metabolic reprogramming and is a potential therapeutic target in pancreatic cancer

Jessica K. Nelson, May Zaw Thin, Theodore Evan, Steven Howell, Mary Wu, Bruna Almeida, Nathalie Legrave, Duco S. Koenis, Gabriela Koifman, Yoichiro Sugimoto, Miriam Llorian Sopena, James MacRae, Emma Nye, Michael Howell, Ambrosius P. Snijders, Andreas Prachalias, Yoh Zen, Debashis Sarker, and Axel Behrens

**Supplementary Figure 1 (related to Figure 1). Characterization of enzymatically active DUBs in PDAC using activity-based proteomics.** (a) In-gel visualization of Cy5-Ub-ABP labelled DUBs in KPCY-organoids. (b) Mass spectrometry analysis of DUBs labelled with Biotin-ubiquitin-ABP in KPCY organoids. Volcano blot depicts the label-free quantification (LFQ) of $-\log_{10}$ t-test p-value vs ratio difference of untreated (right side) and DUBi-treated (left side) samples. (c) Volcano plot shows the significance of each gene set from the Gene Ontology (GO) biological Process 2021 dataset versus its odds ratio from mass spectrometry data in Figure 1c. Each point represents a single geneset; the x-axis measures the odds ratio (0, inf) calculated for the gene set, while the y-axis gives the -log(p-value) of the gene set. Larger blue points represent significant terms (p-value < 0.05); smaller grey points represent non-significant terms. The darker the blue colour of a point, the more significant it is. (d) Bar chart shows the top 10 enriched terms in the GO Biological Process 2021 library, along with their corresponding p-values. (e) Mass spectrometry analysis of DUBs labelled with Biotin-ubiquitin-ABP in KPCY tumor tissue, with or without DUBi treatment (n=3 biologically independent samples per group). Displayed as median normalized $-\log_{10}$ t-test p-value vs ratio difference of untreated (right side) and DUBi-treated (left side) samples. For (b and e) grey dots indicate all proteins identified, and red dots highlight all significant DUBs. Source data are provided as a Source Date file.

**Supplementary Figure 2 (related to Figure 2). Genetic knock-down screen identifies Usp25 as an essential DUB in PDAC organoids.** (a) Representative images of KPCY organoids following puromycin selection with non-targeting (NT) or YFP-targeting shRNAs. Scale bar is 800 μM. (b) Quantification of YFP signal from (a), shown as mean fluorescent intensity (MFI), and displayed as mean ± SD. Statistical

significance was determined by two-sided Student's t-test (n=6 biologically independent experiments). (c) Quantification of organoid growth calculated by % well confluency from live-cell phenotypic monitoring system, and displayed as mean ± SD. Statistical significance was determined by two-sided Student's t-test (n=6 biologically independent experiments). (d) Organoid viability was measured after 72 hours post puromycin selection and shown as relative luminescent unit (RLU), and displayed as mean ± SD. Statistical significance was determined by two-sided Student's t-test (n=6 biologically independent experiments). (e) Representative images from live-cell phenotypic monitoring time-course in KPCY organoids. Confluency mask is shown in yellow. Scale bar is 1 mm. (f) Quantitative values are plotted from two representative control shRNA lines are shown, with a peak growth confluency between 72-90 hours, highlighted with red dashed bars in (f), and red box in (e). Black dashed lines highlight 24- and 114-hour images in (e). Source data are provided as a Source Date file.

**Supplementary Figure 3 (related to Figure 3). USP25 is highly expressed and enzymatically active in PDAC compared to normal pancreatic tissue, which correlated with poor patient survival.** (a) Histological and immunohistochemical analysis of Usp25 expression in normal pancreatic and KPCY tumor tissues. Scale bar is 100 μm. Each image comes for a biologically independent animal, quantified in Figure 3b (b) Histological and immunohistochemical analysis of USP25 staining in primary patient PDAC tumors. Scale bar is 1000 μm and insert is 100 μm. Each image comes from an independent patient.

**Supplementary Figure 4 (related to Figure 4). Depletion of *USP25* leads to reduced patient-derived organoid formation, viability and attenuated PDAC**

**tumor growth *in vivo.*** (a) Representative brightfield images of sh*Usp25* silencing in KPCY organoids. Scale bar is 800 µm. (b) Expression of *Usp25* in KPCY organoids and displayed as relative mRNA expression compared to shYFP controls. Data shown as mean ± SD, and statistical significance was determined by One-way ANOVA with Dunnett post-hoc test (n=4 biologically independent samples). (c) Expression of *USP25* in PDO lines and displayed as relative mRNA expression compared to shNT controls. Data shown as mean ± SD, and statistical significance was determined by One-way ANOVA with Dunnett post-hoc test (n=4 biologically independent samples). (d) Representative images of sh*USP25* silencing in PDOs incubated with caspase-3 activity probe. Scale bar is 800 µm. (e) Schematic of *USP25* knock-out strategy using CRISPR/Cas9. (f) Sanger sequencing data of the USP25 locus from PDO line (K17T) parental and *USP25* knock-out clones. (g) Representative images of the Hu-PDAC organoids, showing empty vector (EV) control and CRISPR/Cas9-mediated *USP25* knock-out cells. Each USP25$^{-/-}$ clones are described with single guide (sg) targeting exon shown. Scale bar is 400 µm. (h) Organoid viability displayed as % relative to the EV, and shown as mean ± SD. Statistical significance was determined by One-way ANOVA with Dunnett post-hoc test (n=3 biologically independent experiments). Source data are provided as a Source Date file.

**Supplementary Figure 5 (related to Figure 5). Transcriptional and metabolomic profiling of PDAC organoids reveals USP25 as a novel regulator of HIF-1 transcriptional activity and metabolic rewiring.** (a) Principal component analysis of log-transformed normalized counts for the top 5000 most variable transcripts (as determined by standard deviation) from the RNA-seq experiment. (b) Hierarchical clustering of log-transformed and mean-centered normalized counts for all

differentially expressed transcripts from the RNA-seq experiment. (c-d) Pathway analysis of differentially expressed genes down-regulated upon loss of *Usp25*. Bar chart shows the top 10 enriched terms in the (c) MsigDB Hallmark 2020 and (d) KEGG 2021 libraries, along with their corresponding p-values. (e-f) Spearman correlation of *USP25* gene expression with gene signatures from (e) the KEGG glycolysis pathway or (f) the HALLMARK hypoxia pathway. Data in (e-f) generated from the GEPIA database, where each dot represents data from one patient expressed as transcripts per million (TPM).

**Supplementary Figure 6 (related to Figure 5). Transcriptional and metabolomic profiling of PDAC organoids reveals USP25 as a novel regulator of HIF-1 transcriptional activity and metabolic rewiring.** (a) Heatmap of gene expression z-scores for the HALLMARK Hypoxia pathway (ID:M5891). (b) Gene expression in KPCY organoids, displayed as relative mRNA expression compared to shYFP controls. Data shown as mean ± SD, and statistical significance was determined by two-sided Students t-test with Holm-Sidak post-hoc correction for multiple testing (n=4 biologically independent experiments). (c) Glucose concentrations measured in culture medium from Mu KPCY organoids, and displayed as mean ± SD. Statistical significance was determined by two-sided Students t-test with Holm-Sidak post-hoc correction for multiple testing (n=3 biologically independent experiments). (d-e) Incorporation of $^{13}$C-glucose into (d) intracellular pyruvate, and (e) TCA metabolites in treated KPCY organoids. Data in (d-e) is displayed as mean ± SD, and represents one experiment carried out with six replicates. Due to technical limitations, statistics for (d-e) were performed on technical replicates using two-sided Student’s t-test. Source data are provided as a Source Date file.

**Supplementary Figure 7 (related to Figure 6). USP25 interacts with, deubiquitylates and stabilizes HIF-1$\alpha$ and promotes its transcriptional activity.** (a) Western blot of indicated targets following siRNA-mediated knock-down of *USP25* or non-targeting (NT) control in human PDAC cell lines, treated with 200 µM cobalt chloride ($CoCl_2$) for six hours. (b) PDOs (line K13T) were treated with the cell-permeable Image-IT Red Hypoxia reagent to measure cellular hypoxia. Scale bar is 100µm. (c) Organoid viability in indicated PDO lines measured at day 5 under high oxygen tension (20%) or low oxygen tension (1%) culture conditions. Values shown as relative luciferase unit (RLU), and displayed as mean ± SD. Statistical significant was determined by two-way ANOVA with post hoc corrections for multiple testing (n= 4 biologically independent experiments). (d) Western blot of indicated targets following shRNA-mediated knock-down of *USP25* or non-targeting control in PDOs (line K13T), with 200 µM $CoCl_2$ treatment for six hours where indicated. Source data are provided as a Source Date file.

**Supplementary Figure 8 (related to Figure 6). USP25 interacts with, deubiquitylates and stabilizes HIF-1$\alpha$ and promotes its transcriptional activity.** (a) HEK293T transfected with the indicated expression constructs for epitope-tagged USP25 and HIF-1$\alpha$. GFP was co-transfected as a transfection efficiency and additional loading control. 24 hours post-transfection, cells were treated for an additional 24 hours with 200 µM $CoCl_2$. Total cell lysates were immunoblotted or immunoprecipitated and analyzed as indicated. (b) Human renal carcinoma cell line RCC-4 was treated for 72 hours with siRNA for non-targeting (siNT) or four individual *USP25* targeting siRNA (*siUSP25* 1-4). Total cell lysates were immunoblotted as

indicated. (c) Western blot of indicated targets following siRNA-mediated knock-down of *USP25* or non-targeting control. (d) HEK293T cells transfected with indicated expression constructs for epitope-tagged HIF-1$\alpha$ and USP25 and treated with 200 µM cobalt chloride ($CoCl_2$) for six hours. Sub-cellular fractionation was done to compare cytosolic and nuclear expression of indicated targets. (e) *SLC2A1* mRNA expression in HEK293T cells, displayed as relative mRNA expression compared to EV transfected control. Data shown as mean ± SD, and statistical significance was determined by One-way ANOVA with Dunnett post-hoc test (n=4 biologically independent experiments). (f) Schematic of the lentiviral pInducer20-blast construct which was used to engineer PDO lines with DOX-inducible USP25 wild-type (WT) or catalytic mutant (C178S) expression. (g) Western blot images of endogenous protein levels following 48 hours of 1 µM DOX treatment in PDOs (line K13T) with DOX-inducible USP25-WT or -C178S expression. (h) Gene expression in PDOs, displayed as relative mRNA expression compared to controls (grey bars). Each bar represents the mean ± SD, and each symbol represents a different shRNA targeting either *HIF1A* or YFP control from the average of three biologically independent PDO lines. Statistical significance was determined by two-sided Students t-test with Holm-Sidak post-hoc correction for multiple testing. (i) Western blot images of protein levels following 72 hours of 500 nM DOX to induce expression of the HA-tagged HIF-1$\alpha$ stabilization mutant (HIF1A$^{P402A/P456A}$) in indicated PDO lines. Source data are provided as a Source Date file.

**Supplementary Figure 9 (related to Figure 7). Pharmacological inhibition of USP25 leads to loss of HIF-1$\alpha$ signaling and reduced tumor growth *in vitro* and *in vivo*.** (a) Brightfield images of Mu wild-type (WT) and KPCY organoids treated with

100nM FT206. Scale bar is 500 µm. (b) Total cell lysates were immunoblotted as indicated. (c) *Usp28* knock-out organoids treated with FT206 at the indicated doses for 72 hours. Organoid viability was displayed as % relative to vehicle-treated control, and shown as mean ± SD. Statistical significance was determined by One-way ANOVA with Dunnett post-hoc test (n=3 biologically independent experiments). (d) Representative brightfield images from (Figure 7c) of PDO lines treated with vehicle or 100nM FT206. Scale bar is 0.5 mm. (e) Gene expression in KPCY organoids, displayed as relative mRNA expression compared to vehicle-treated controls. Data shown as mean ± SD, and statistical significance was determined by two-sided Student's t-test with Holm-Sidak post-hoc correction for multiple testing (n=4 biologically independent experiments). (f) Heatmap displaying organoid viability in KPCY organoids treated with FT206 or different tankyrase and Wnt signaling inhibitors at indicated concentrations, displayed as relative luciferase unit (RLU). (g) Heatmap displaying organoid viability of PDO lines treated AZ6102 with indicated concentrations, calculated as % of vehicle-treated. Source data are provided as a Source Date file.

**Supplementary Figure 10 (related to Figure 7). Pharmacological inhibition of USP25 leads to loss of HIF-1α signaling and reduced tumor growth *in vitro* and *in vivo*.** (a) Subcutaneous tumor weights at endpoint from experiments derived with different KPCY organoid lines. Data displayed as mean ± SD, and statistical significance was determined by two-sided Student's t-test (KPCY-1 is equal to n=6 biologically individual animals per group, and KPCY-2 and -3 are equal to n=5 biologically individual animals per group). (b) Body weights of animals treated with FT206 (75 mg/kg) or vehicle, three times a week for 5 weeks. Data plotted as mean ±

SD (n=5 biologically individual animals per group). (c) Growth curves of PDOX volumes (line K3T), treated with vehicle or FT206. Data plotted as mean ± SD, and statistical significance was determined by Two-way ANOVA (n=5 biologically individual animals per group). (d) Representative images of 3D ultrasound images of PDOXs described in (c). Scale bar is 3mm (e) Endpoint tumor volumes ($mm^3$) of PDOX, from lines K3T (n=5 biologically individual animals per group) and K10T (n=6 biologically individual animals per group). Statistical significance was determined by two-sided Student's t-test. (f) Body weights of animals treated with FT206 (75 mg/kg) or vehicle, two-three times a week for 6 weeks. Data plotted as mean ± SD (n=5 biologically individual animals per group). (g) Representative immunohistochemical analysis of active cleaved caspase-3 (C3A) expression in subcutaneous KPCY organoid tumors. Scale bar is 1000 µm. Quantification shown in Figure 7h. Source data are provided as a Source Date file.

**Supplementary Figure 11 (related to Figure 7). Pharmacological inhibition of USP25 leads to loss of HIF-1α signaling and reduced tumor growth *in vitro* and *in vivo*.** (a) Representative images of immunofluorescent staining of DAPI (grey), CK19 (blue) and hypoxyprobe (red) in PDOX treated with vehicle or FT206. Scale bar is 1000 µm, and insert is 100 µm. (b) Quantification of hypoxyprobe positive staining area in whole tumor sections. Data displayed as mean ± SD, and statistical significance was determined by two-sided Student's t-test (n=14 biologically independent animals for vehicle and n=10 biologically independent animals for FT206 groups). (c) Representative images of immunofluorescent staining of Slc2a1 (red) and Lectin (blue) in KPCY subcutaneous tumors treated with vehicle or FT206. Scale bar is 1000 µm, and insert b and d are 100 µm. (d) Quantification of Slc2a1 positive

staining area, displayed as mean ± SD. Statistical significance was determined by two-sided Student’s t-test (n=8 biologically independent animals per group). (e) Quantification of Lectin positive staining area, displayed as mean ± SD. Statistical significance was determined by two-sided Student’s t-test (n=8 biologically independent animals per group). (f) Representative images of subcutaneous tumors of KPCY organoids treated with vehicle or FT206. (g) Immunohistochemical analysis of SLC2A1 staining in primary patient PDAC tumors. Scale bar is 50 μm. (h) Correlation between protein expression of SLC2A1 (% positive area) versus USP25 (% positive area) was assessed using linear regression. R-value and p-value are shown in graph inset. Source data are provided as a Source Date file.

**Supplementary Figure 12. Graphical summary.**

USP25 is highly expressed and active in PDAC, compared to normal pancreatic tissue. USP25 antagonizes polyubiquitination of HIF-1α, leading to HIF-1α stabilization and transcriptional activity. Enhanced HIF-1α transcriptional activity promotes metabolic reprogramming and tumor cell survival in the hypoxic tumor microenvironment.

**Supplementary Table 1.** Patient-derived organoids used in this study with detailed description of morphology, mutational status and primary tumour stage and histology.

| PDO Line | PDO morphology | KRAS status | TP53 status | TNM Stage | Histology | Neoadjuvant Chemo? |
|---|---|---|---|---|---|---|
| K3T | non-cystic | missense mutation | missense mutation | T2N1M0 | Poorly differentiated | None |
| K4T | cystic | missense mutation | missense mutation | T2N2M0 | Moderately differentiated | None |
| K5T | non-cystic | missense mutation | no mutation | T3N2M0 | Moderately differentiated | None |
| K7T | cystic | missense mutation | deep deletion | T4N1M0 | Moderately differentiated | FOLFIRINOX 12 Cycles |
| K10T | non-cystic | missense mutation | missense mutation | T2N1M0 | Moderately differentiated | None |
| K13T | cystic | missense mutation | no mutation | T2N1M0 | Moderately differentiated | None |
| K17T | cystic | missense mutation | no mutation | Unknown | Moderately differentiated | None |
| K19T | cystic | missense mutation | nonsense mutation | Unknown | Moderately differentiated | None |
| K22T | non-cystic | missense mutation | missense mutation | T2N1M0 | Moderately differentiated | None |
| K25T | non-cystic | missense mutation | missense mutation | T2N1M0 | Moderately differentiated | None |
| K28T | cystic | missense mutation | missense mutation | T2N1M0 | No diff. status | None |
| K36T | non-cystic | n/a | n/a | T3N1M0 | Adenosquamous | None |
| K38T | cystic | missense mutation | n/a | T2N1M0 | No diff. status | None |
| K46T | non-cystic | missense mutation | n/a | T2N2M0 | Moderately differentiated | None |

**Supplementary Table 2.** shRNA target sequences.

| Gene name | Species | Clone ID | Sequence (5'-3') |
|---|---|---|---|
| YFP-1 | N/A | N/A | ccgggaacggcatcaaggtgaacttctcgagaagttcaccttgatgccgttctttttg |
| YFP-2 | N/A | N/A | ccggcaacagccacaacgtctatatctcgagatatagacgttgtggctgttgtttttg |
| YFP-3 | N/A | N/A | ccggggagcgcaccatcttcttcaactcgagttgaagaagatggtgcgctcctttttg |
| Non-Target-1 | N/A | SHC002 | ccggcaacaagatgaagagcaccaactcgagttggtgctcttcatcttgttgtttttg |
| Non-Target-2 | N/A | SHC0016 | ccgggcgcgatagcgctaataatttctcgagaaattattagcgctatcgcgctttttg |
| Usp8-1 | Mu | TRCN0000030744 | ccggcctcacatctaatgcttacaactcgagttgtaagcattagatgtgaggtttttg |
| Usp8-2 | Mu | TRCN0000030745 | ccggcgcagtttacaaccaatgctactcgagtagcattggttgtaaactgcgtttttg |
| Usp8-3 | Mu | TRCN0000030746 | ccggccaaaggattaacggcgagaactcgagttctcgccgttaatcctttggtttttg |
| Usp14-1 | Mu | TRCN0000030758 | ccgggagaagtttgaaggtgtagaactcgagttctacaccttcaaacttctctttttg |
| Usp14-2 | Mu | TRCN0000030757 | ccgggcagctcttagagatttgtttctcgagaaacaaatctctaagagctgctttttg |
| Usp14-3 | Mu | TRCN0000312005 | ccgggagaagtttgaaggtgtagaactcgagttctacaccttcaaacttctctttttg |
| Uchl3-1 | Mu | TRCN0000030714 | ccggtcttgacagaaacaccaaatactcgagtatttggtgtttctgtcaagatttttg |
| Uchl3-2 | Mu | TRCN0000030715 | ccggcctgaacttcttagcatggtactcgagtaccatgctaagaagttcaggtttttg |
| Uchl3-3 | Mu | TRCN0000030718 | ccgggcatggtaccaagaccagtatctcgagatactggtcttggtaccatgctttttg |
| Usp19-1 | Mu | TRCN0000030782 | ccggcgatcctttgaagctgagattctcgagaatctcagcttcaaaggatcgtttttg |
| Usp19-2 | Mu | TRCN0000030780 | ccggcggcacaagatgagaaatgatctcgagatcatttctcatcttgtgccgtttttg |
| Usp19-3 | Mu | TRCN0000335553 | ccggcggcacaagatgagaaatgatctcgagatcatttctcatcttgtgccgtttttg |
| Usp48-1 | Mu | TRCN0000086675 | ccggccgaattgcttggttggcattctcgagaatgccaaccaagcaattcggtttttg |
| Usp48-2 | Mu | TRCN0000086673 | ccgggctgtcaacaatttgctgaaactcgagtttcagcaaattgttgacagctttttg |
| Usp48-3 | Mu | TRCN0000086677 | ccggctgaaggaagaacgattagaactcgagttctaatcgttcttccttcagtttttg |
| Usp7-1 | Mu | TRCN0000087152 | ccgggccgaatttaacagagagaatctcgagattctctctgttaaattcggctttttg |
| Usp7-2 | Mu | TRCN0000087150 | ccggcctgcaatgttagataatgaactcgagttcattatctaacattgcaggtttttg |
| Usp7-3 | Mu | TRCN0000087151 | ccgggcaacttatgaggttcatgtactcgagtacatgaacctcataagttgctttttg |
| Otud6b-1 | Mu | TRCN0000113316 | ccgggccagagaattggaaattaaactcgagtttaatttccaattctctggctttttg |
| Otud6b-2 | Mu | TRCN0000113318 | ccggccagacatcttgagagtgaaactcgagtttcactctcaagatgtctggtttttg |
| Otud6b-3 | Mu | TRCN0000113319 | ccgggctgagtacatgcaaacccatctcgagatgggtttgcatgtactcagctttttg |
| Usp28-1 | Mu | TRCN0000030868 | ccgggctgttattcagtctctctttctcgagaaagagagactgaataacagctttttg |

| | | | |
|---|---|---|---|
| Usp28-2 | Mu | TRCN0000030865 | ccggcggaagttgaaggaggaaatactcgagtatttcctccttcaacttccgtttttg |
| Usp28-3 | Mu | TRCN0000030866 | ccgggcagtacattcaggaagataactcgagttatcttcctgaatgtactgctttttg |
| Usp15-1 | Mu | TRCN0000231399 | ccggtgagaggtgaaatagctaaatctcgagatttagctatttcacctctcatttttg |
| Usp15-2 | Mu | TRCN0000231397 | ccggcagactgtggaacaagtatatctcgagatatacttgttccacagtctgtttttg |
| Usp15-3 | Mu | TRCN0000033215 | ccgggctgacacaatagatacgattctcgagaatcgtatctattgtgtcagctttttg |
| Uchl5-1 | Mu | TRCN0000030724 | ccgggcaatcaagatgactggattactcgagtaatccagtcatcttgattgctttttg |
| Uchl5-2 | Mu | TRCN0000030726 | ccgggtacgcatcaagatgtgcattctcgagaatgcacatcttgatgcgtactttttg |
| Uchl5-3 | Mu | TRCN0000030725 | ccggcttgcctttcattatggaattctcgagaattccataatgaaaggcaagtttttg |
| Otud7b-1 | Mu | TRCN0000311256 | ccggggtagatattagggttgaatactcgagtattcaaccctaatatctacctttttg |
| Otud7b-2 | Mu | TRCN0000308627 | ccgggcagaaggaatggaatgaattctcgagaattcattccattccttctgctttttg |
| Otud7b-3 | Mu | TRCN0000308626 | ccggcctttagtggagggagtacttctcgagaagtactccctccactaaaggtttttg |
| Otub1-1 | Mu | TRCN0000030982 | ccgggagcaagttcttcgagcacttctcgagaagtgctcgaagaacttgctctttttg |
| Otub1-2 | Mu | TRCN0000030983 | ccgggtccatccaagtggagtacatctcgagatgtactccacttggatggactttttg |
| Otub1-3 | Mu | TRCN0000335250 | ccgggagcaagttcttcgagcacttctcgagaagtgctcgaagaacttgctctttttg |
| Usp5-1 | Mu | TRCN0000030738 | ccggcgaggatgtgaagattgtcatctcgagatgacaatcttcacatcctcgtttttg |
| Usp5-2 | Mu | TRCN0000030737 | ccggcctgggctacatctacttctactcgagtagaagtagatgtagcccaggtttttg |
| Usp5-3 | Mu | TRCN0000030736 | ccggcgaatgttcaaggccctcattctcgagaatgagggccttgaacattcgtttttg |
| Usp24-1 | Mu | TRCN0000040628 | ccgggctggattctttaggcagaaactcgagtttctgcctaaagaatccagctttttg |
| Usp24-2 | Mu | TRCN0000086746 | ccggcccgagctcttgtctgccattctcgagaatggcagacaagagctcgggtttttg |
| Usp24-3 | Mu | TRCN0000086745 | ccggtcattggtctatcccgtacaactcgagttgtacgggatagaccaatgatttttg |
| Usp9x-1 | Mu | TRCN0000030759 | ccggcggcttaactttcttaggtttctcgagaaacctaagaaagttaagccgtttttg |
| Usp9x-2 | Mu | TRCN0000030763 | ccggcctcaacaagtttggcactttctcgagaaagtgccaaacttgttgaggtttttg |
| Usp9x-2 | Mu | TRCN0000030761 | ccgggcagaagaaatcactatgattctcgagaatcatagtgatttcttctgctttttg |
| Atxn3-1 | Mu | TRCN0000123962 | ccgggctcagaattgatcctataaactcgagtttataggatcaattctgagctttttg |
| Atxn3-2 | Mu | TRCN0000123963 | ccggctcgcactattcttggctcaactcgagttgagccaagaatagtgcgagtttttg |
| Atxn3-3 | Mu | TRCN0000123961 | ccgggctcactttgtgctcagcattctcgagaatgctgagcacaaagtgagctttttg |
| Otud4-1 | Mu | TRCN0000252253 | ccggccgtgtcacaagcgcatttaactcgagttaaatgcgcttgtgacacggtttttg |
| Otud4-2 | Mu | TRCN0000252254 | ccgggcgtttatagaagggtcatttctcgagaaatgacccttctataaacgctttttg |
| Otud4-3 | Mu | TRCN0000252251 | ccggcacgttagattggatcataatctcgagattatgatccaatctaacgtgtttttg |

| | | | |
|---|---|---|---|
| Usp25-1 | Mu | TRCN0000030827 | ccggcccaacgatcactgcaagaaactcgagtttcttgcagtgatcgttgggtttttg |
| Usp25-2 | Mu | TRCN0000233427 | ccggttatatctggacaggtatatgctcgagcatatacctgtccagatataatttttg |
| Usp25-3 | Mu | TRCN0000233428 | ccggcatcgctggaggacggaaatactcgagtatttccgtcctccagcgatgtttttg |
| Usp25-4 | Mu | TRCN0000233431 | ccggtagtataatggaaccatattgctcgagcaatatggttccattatactatttttg |
| USP25-1 | Hu | TRCN0000004366 | ccgggcacttctcctgttgacgatactcgagtatcgtcaacaggagaagtgcttttt |
| USP25-2 | Hu | TRCN0000004368 | ccgggctgttcctcatctgtgcttactcgagtaagcacagatgaggaacagcttttt |
| USP25-3 | Hu | TRCN0000004369 | ccgggcgtgagctgaggtatctattctcgagaatagatacctcagctcacgcttttt |
| USP25-4 | Hu | TRCN0000004370 | ccggtggaggagtaagatgaaatatctcgagatatttcatcttactcctccattttt |
| HIF1A-1 | Hu | TRCN0000003808 | ccggccgctggagacacaatcatatctcgagatatgattgtgtctccagcggtttttt |
| HIF1A-2 | Hu | TRCN0000003811 | ccggcggcgaagtaaagaatctgaactcgagttcagattctttacttcgccgtttttt |
| HIF1A-3 | Hu | TRCN0000010819 | ccggtgctctttgtggttggatctactcgagtagatccaaccacaaagagcattttt |

**Supplementary Table 3.** RT-qPCR primers.

| Target | Species | FWD sequence (5'-3') | REV sequence (5'-3') |
|---|---|---|---|
| 36b4 | Mu/hu | gtgctgatgggcaagaac | aggtcctccttgtgaac |
| Tbp | Mu | cggtcgcgtcattttctc | gggttatcttcacacacca |
| Tbp | Hu | ccactcacagactctcacaac | ctgcggtacaatcccagaact |
| Atxn3 | Mu | ggagacgagaagcctactttga | tggatatgaaaggggtccag |
| Otub1 | Mu | gggtagcgactccgaagg | gattctgcacagcaatctcttg |
| Otud4 | Mu | gatgccctcacagatcacaa | tggggagaggccaaaatagt |
| Otud6b | Mu | agaaggcatcggaaggaga | agcgttcttcattccctgaa |
| Otud7b | Mu | cggggttctgctcaaaag | tcatgggatcatcaggtactctc |
| Uchl3 | Mu | agcagtcatggagggtcaa | agcagtcatggagggtcaa |
| Uchl5 | Mu | gccaggagaagaacctgct | cctgagtggcacaagcatta |
| Usp5 | Mu | ttcacctttggcttagactgg | gagctcctctggcatctcaa |
| Usp7 | Mu | cccgaggacatggagatg | cattgatgacagggttctgagta |
| Usp8 | Mu | caaaaagagacctgatttcaagc | tgatgtttgcaggtccaagt |
| Usp9x | Mu | tagtccgaggatctgccagt | aaaccgcacgttcttgct |
| Usp14 | Mu | ggcgaacaagggcagtatc | tctgttgcaggactctcatca |
| Usp15 | Mu | gctgtttccaaccactatggag | ccacttcccatcatctttgttt |
| Usp19 | Mu | ggaaccggaatcgagagc | cttcagcaaagaaccaagagc |
| Usp24 | Mu | cgagtctaccaaagataccttcact | gacgctgcctatcgtctcat |
| Usp25 | Mu | cagtcggtctgaagaatgtcg | aactccaaaagattgaataatgactg |
| Usp28 | Mu | ctgcttccttcggatcgtt | cactggaggcagctttgtaa |
| Usp48 | Mu | agactcgaaactctgtcaacctt | gctgggccatatgagagcta |
| USP25 | Hu | tcagagacttctgtgacaacagc | tcacttcttgatggctgctcta |
| HIF1A | Hu | ttttcaagcagtaggaattggaa | gtgatgtagtagctgcatgatcg |
| HIF1B | Hu | ctgtcatcctgaagaccagcag | ctggttctcatccagagccattc |
| HIF2 | Hu | ctgtgtctgagaagagtaacttcc | ttgccataggctgaggactcct |
| MYC | Hu | tctccttgcagctgcttag | gtcgtagtcgaggtcatag |
| ENO1 | Hu | tcccaacatcctggagaataa | atgccgatgaccaccttatc |
| ENO3 | Hu | ttgagaagaaggcctgcaa | cccccagccattagactg |
| HK1 | Hu | tgaggttggactcattgttgg | ccaccatctccacgttcttc |
| LDHA | Hu | cgtcagcaagagggagaaag | gccacgtaggtcaagatatcc |
| PFKL | Hu | acacccgtgtaactgtgctg | atgcccatcttgctgctc |
| PKM | Hu | cagccaaaggggactatcct | caaataattgcaagtggtagatgg |
| SERPINE1 | Hu | aaggcacctctgagaacttca | cccaggactaggcaggtg |
| SLC2A1 | Hu | ggttgtgccatactcatgacc | gagataggacatccagggtagc |
| Hif1a | Mu | catgatggctccctttttca | gtcacctggttgctgcaata |
| Eno1 | Mu | gaggcgcttagtgctgct | atagacatggcgaatttctgg |
| Eno3 | Mu | gaggggtgaactgacactg | gagtcttctccacccgaaga |
| Hk1 | Mu | gtggacgggacgctctac | ttcactgtttggtgcatgatt |
| Ldha | Mu | gctccccagaacaagattacag | tcgcccttgagtttgtcttc |
| Pfkl | Mu | attgaccggcatggaaag | aagcccagcctctgaacc |
| Pfkp | Mu | gaggaccccatctgcat | gtagcttccagcaaggcaat |
| Pkm | Mu | gcagcagctttgatagttctcag | tcgagtcacggcaatgatag |
| Serpine1 | Mu | aggatcgaggtaaacgagagc | gcgggctgagatgacaaa |
| Slc2a1 | Mu | gaccctgcacctcattgg | gatgctcagataggacatccaag |

**Supplementary Table 4.** Electroporation settings for induction of PDO lines using the NEPA21 super electroporator from Nepagene.

| Parameter | Poring Pulse | Transfer Pulse |
|---|---|---|
| Voltage | 175V | 20V |
| Pulse Length | 5.0ms | 50ms |
| Pulse Interval | 50.0ms | 50ms |
| Number of Pulses | 2 | 5 |
| Decay Rate | 10% | 40% |
| Polarity | + | +/- |

**Supplementary Table 5.** Antibodies used for immunohistochemistry, immunofluorescence, and western blotting.

| ANTIBODY | Company, product # | Dilution | Application |
|---|---|---|---|
| Rabbity anti USP25 | Sigma-Aldrich; HPA024142 | 1:100x | IHC |
| Rabbit anti SLC2A1 (Glut1) | Alpha Diagnostics, GT11-A | 1:100x | IHC |
| Rabbity anti Caspase 3 | R&D Bio-Techne, AF825 | 1:100x | IHC |
| Rat anti CK19 | Developmental Studies Hybridoma Bank, TROMA-III | 1:100x | IHC |
| Goat anti GFP | Abcam, ab6673 | 1:100 | IHC |
| Rabbit anti SLC2A1 (Glut1) | Alpha Diagnostics, GT11-A | 1:100x | IF |
| Rat anti CK19 | Developmental Studies Hybridoma Bank, TROMA-III | 1:100x | IF |
| Mouse anti αSMA | Sigma-Aldrich, A5228 | 1:100x | IF |
| Donkey anti rat IgG Alexa Fluor488 | Life Technology, A21208 | 1:200x | IF |
| Donkey anti mouse IgG Alexa Fluor546 | Life Technology, A10036 | 1:200x | IF |
| Donkey anti rabbit IgG Alexa Fluor647 | Life Technology, A31573 | 1:200x | IF |
| Mouse anti β-ACTIN | Abcam, ab49900 | 1:10000x | WB |
| Rabbit anti GAPDH | Abcam, ab9485 | 1:5000 | WB |
| Goat anti GFP | Abcam, ab6673 | 1:2000 | WB |
| Rabbit anti Histone 3B | Millipore, 06755 | 1:1000 | WB |
| Rabbit anti HIF-1α | Novus, NB100-449 | 1:1000x | WB |
| Mouse anti FLAG-HRP M2 | Sigma-Aldrich, A8592 | 1:10000 | WB |
| Rabbit anti HIF-2 | Cell Signaling, 7096 | 1:1000 | WB |
| Rabbit anti ARNT (HIF-1β) | Cell Signaling, 5537 | 1:1000 | WB |
| Rabbit anti HA | Sigma-Aldrich, H6908 | 1:1000 | WB |
| Rabbit anti SLC2A1(GLUT1) | Alpha Diagnostics, GT11-A | 1:1000 | WB |
| Rabbit anti c-Myc | Abcam, Y69 clone, ab32072 | 1:1000 | WB |
| Rabbit anti USP25 | Abcam, ab187156 | 1:2000 | WB |
| Rabbit anti USP28 | Sigma, HP006779 | 1:1000 | WB |
| Mouse anti V5 (tag) | Invitrogen, R960-25 | 1:5000 | WB |
| Rabbit anti VHL | Cell Signaling, 68547 | 1:1000 | WB |

# Supplementary Figure 1

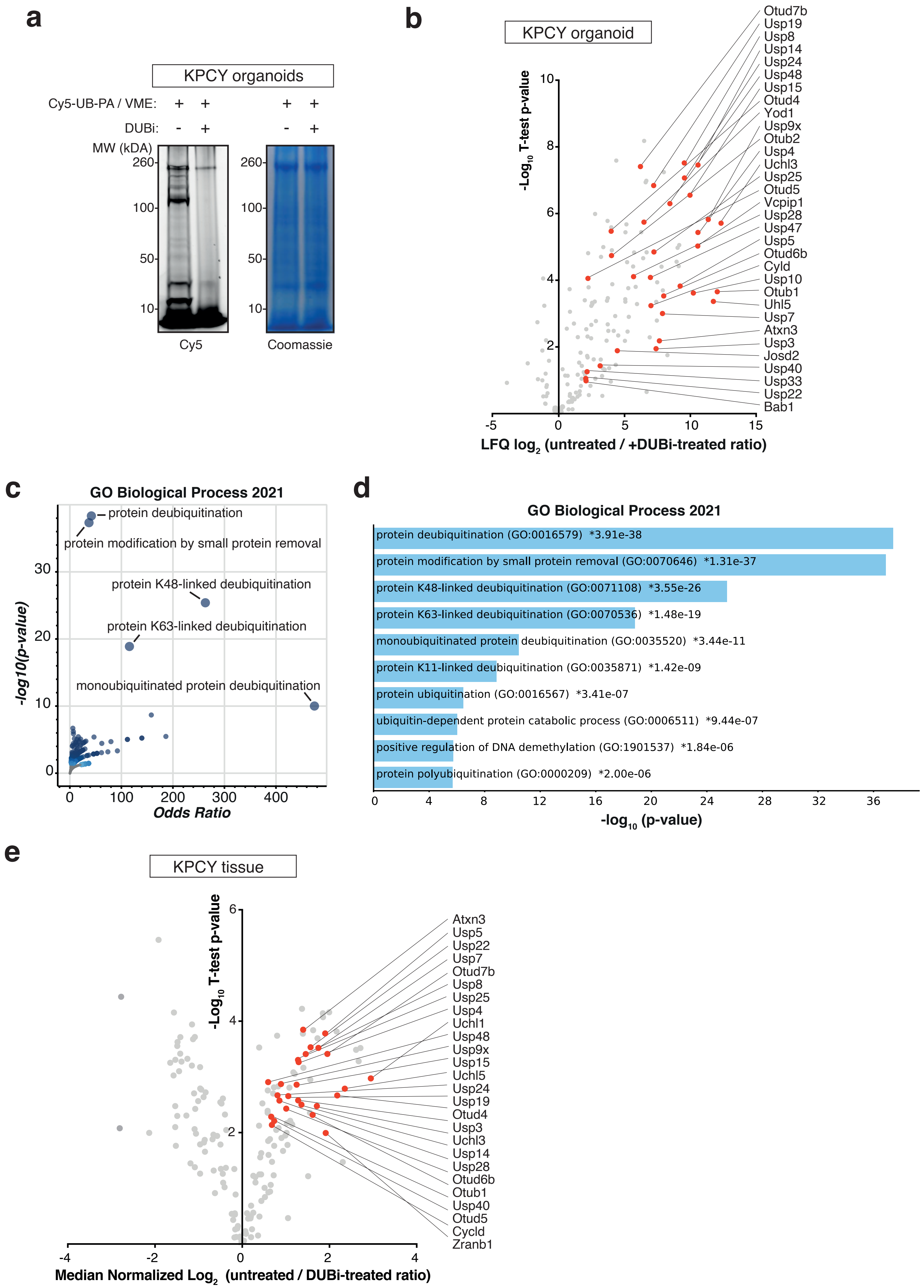

# Supplementary Figure 2

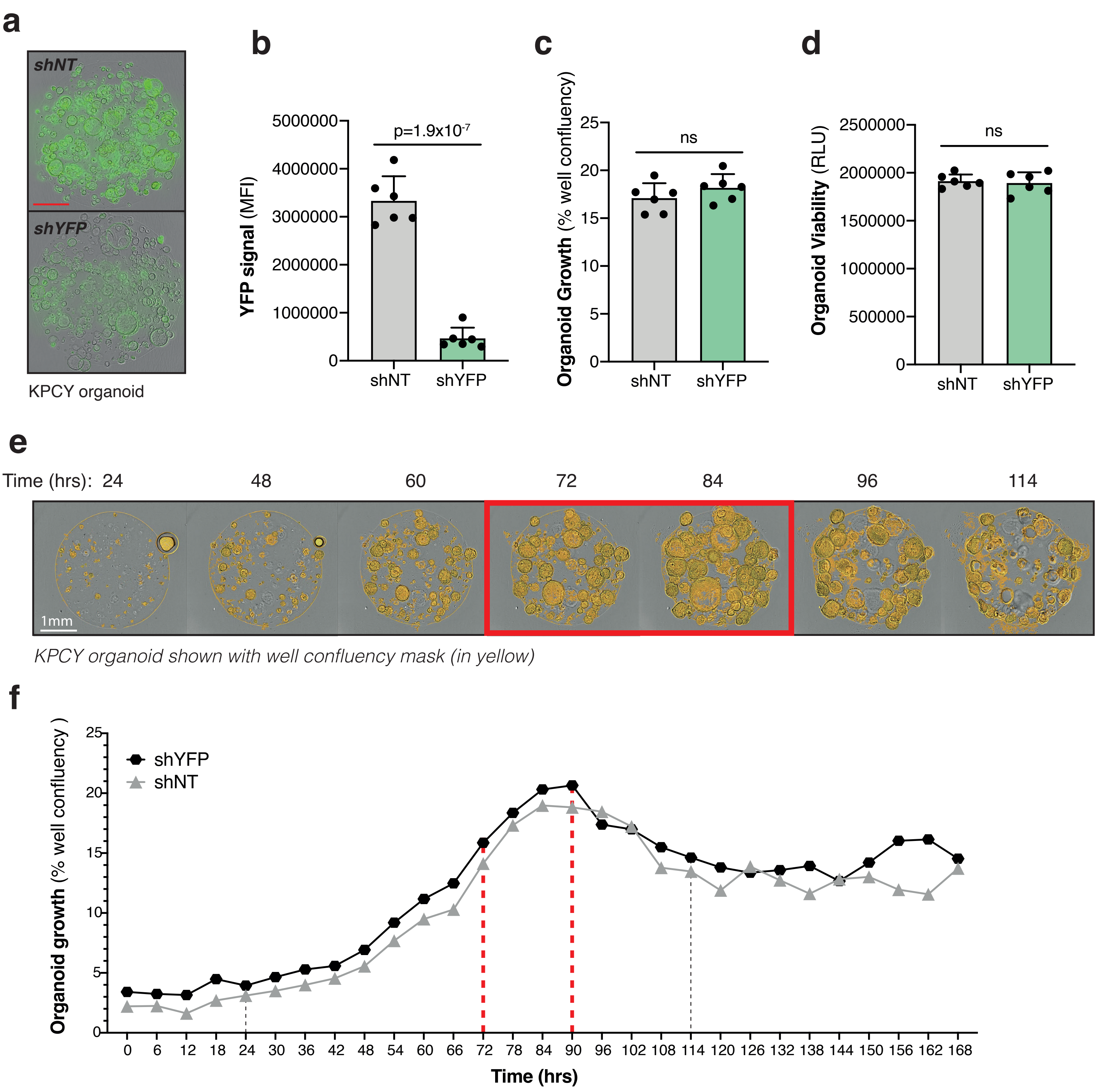

# Supplementary Figure 3

**a**

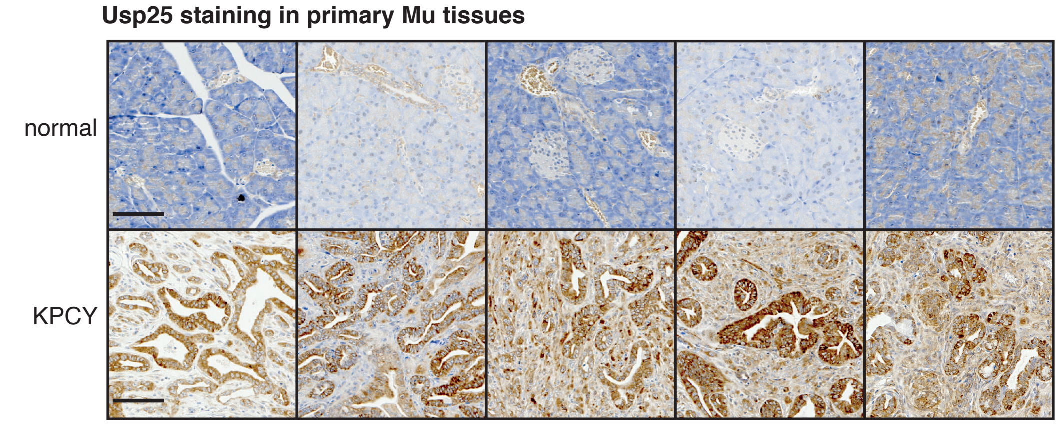


**b**

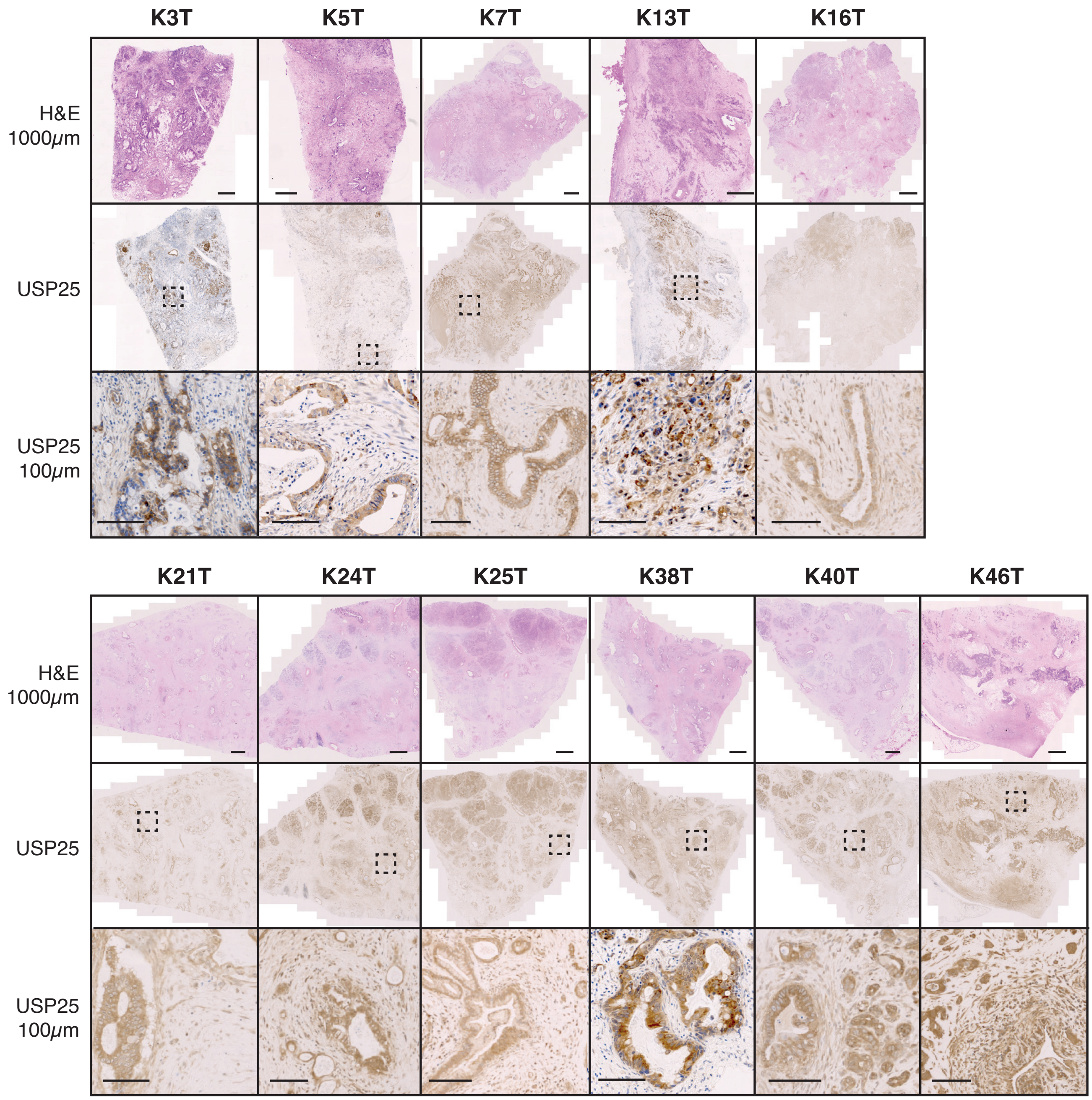


***human PDAC tissue***

# Supplementary Figure 4

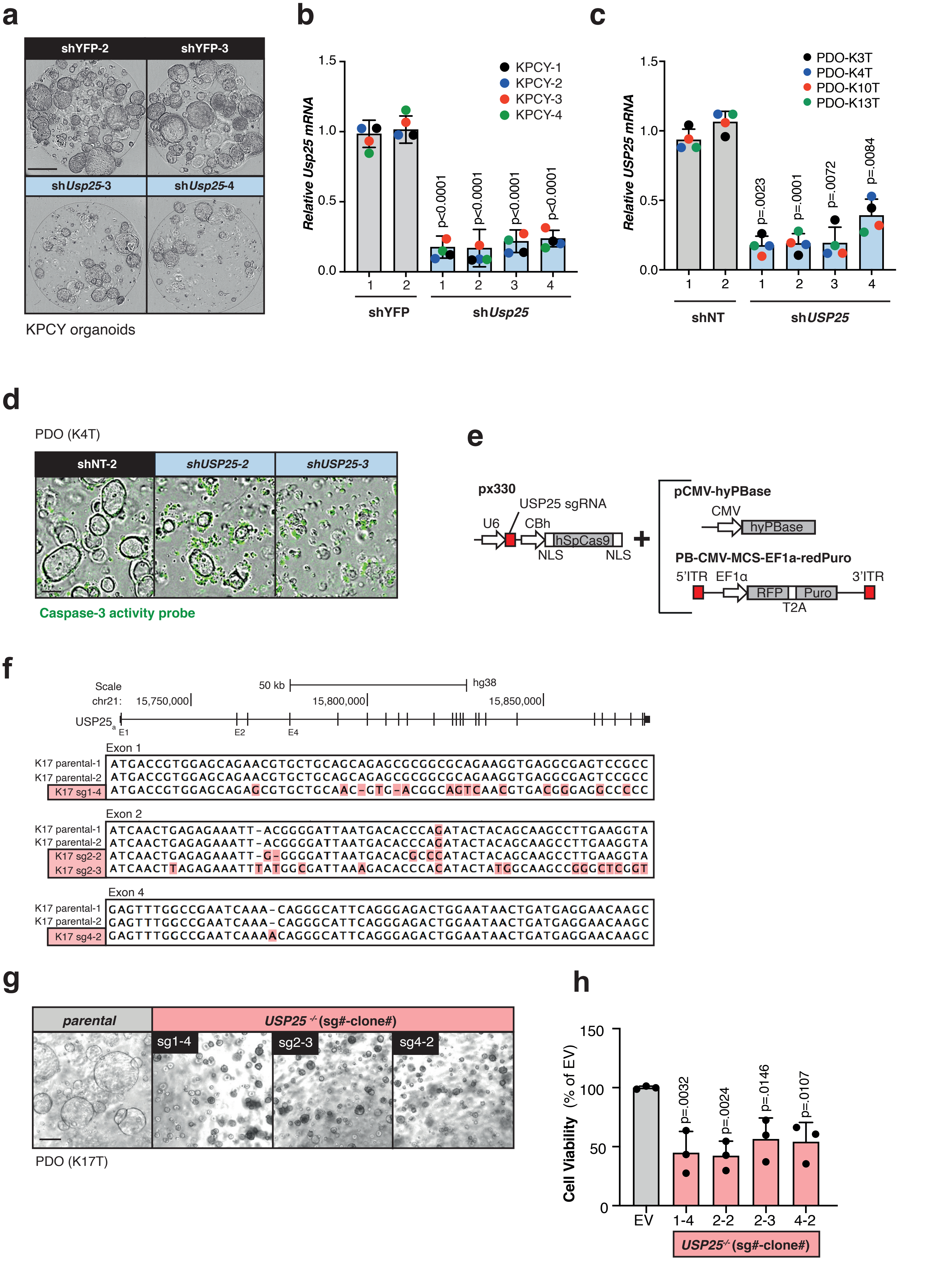

# Supplementary Figure 5

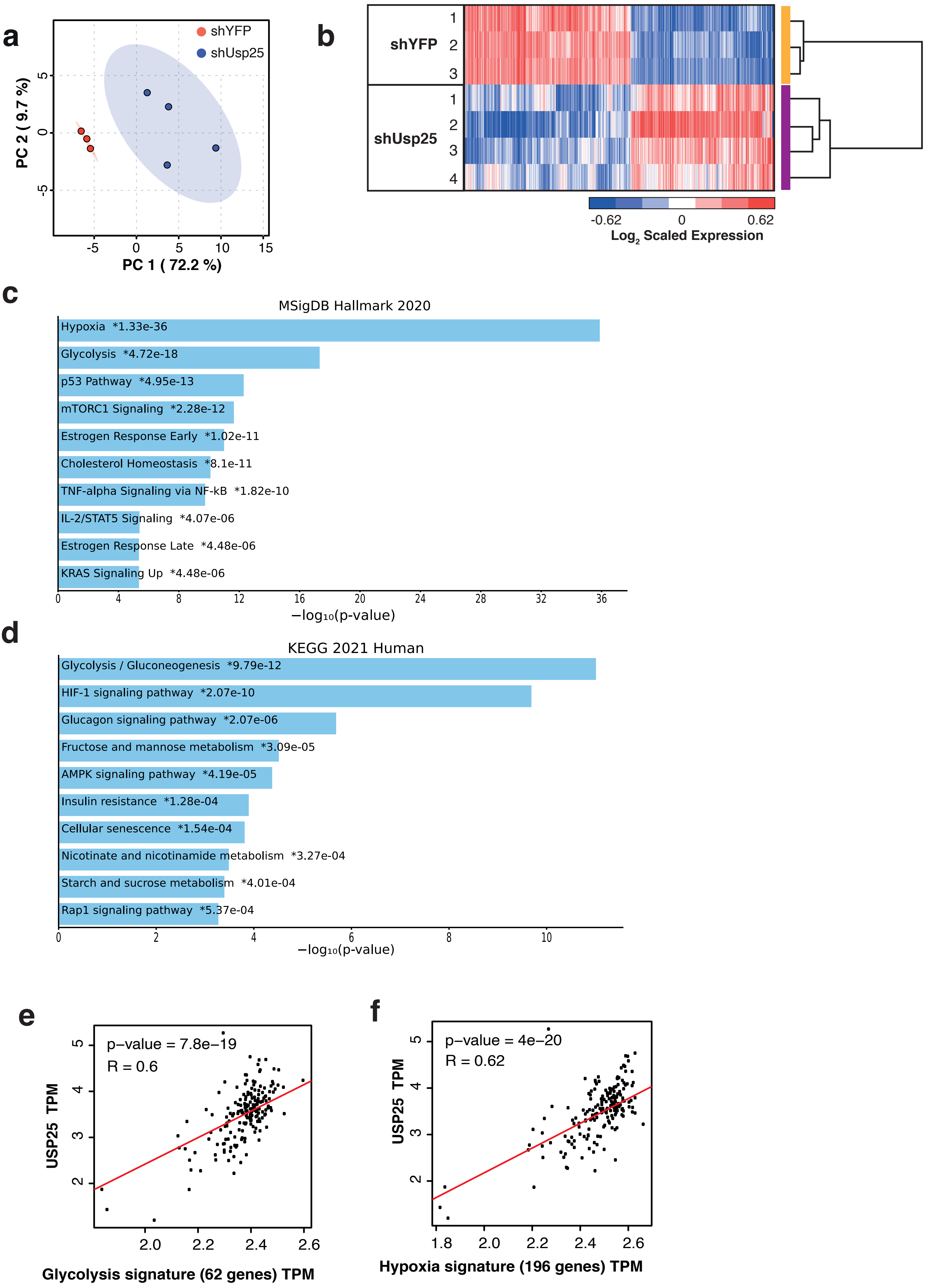

# Supplementary Figure 6

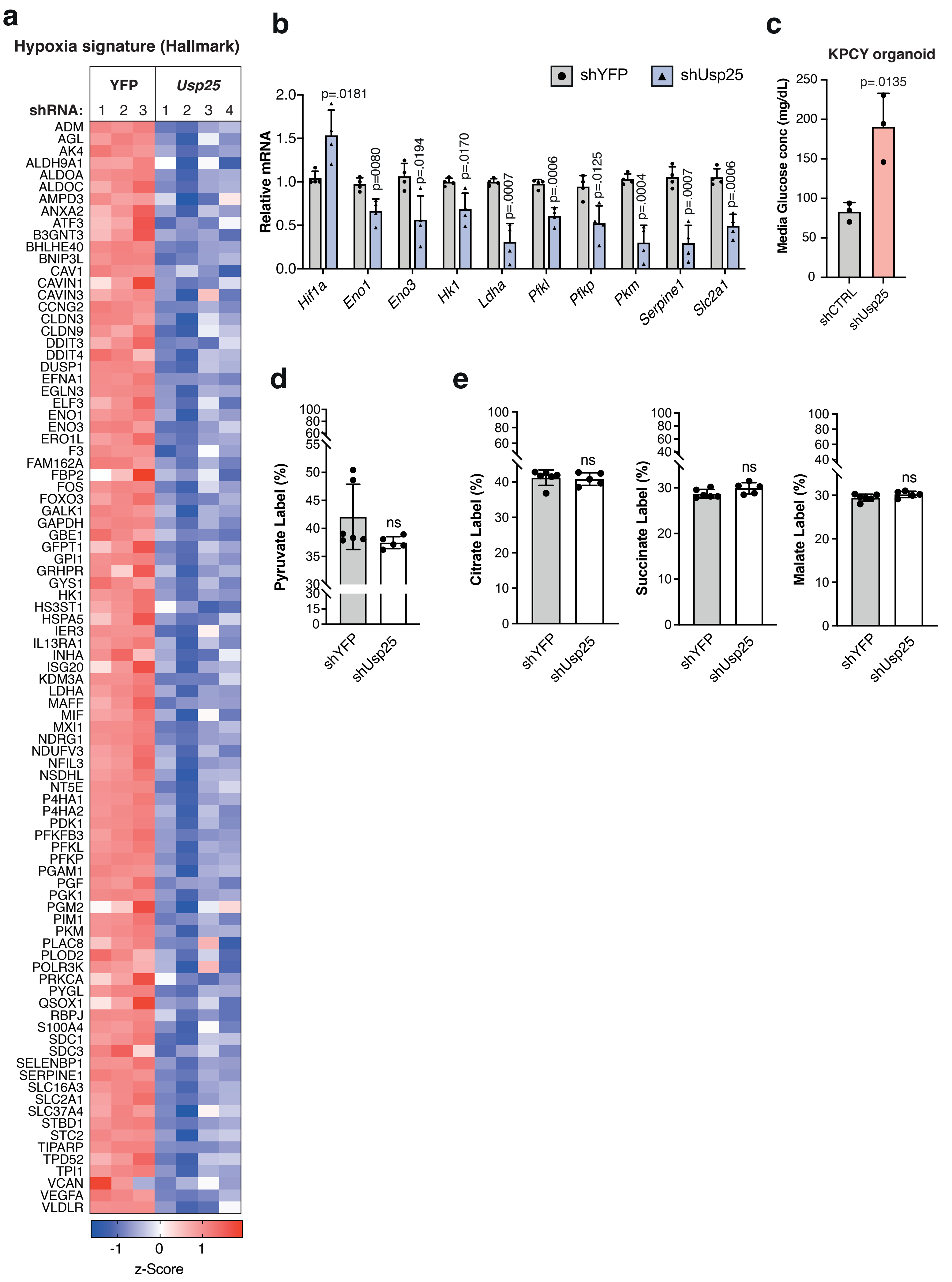

# Supplementary Figure 7

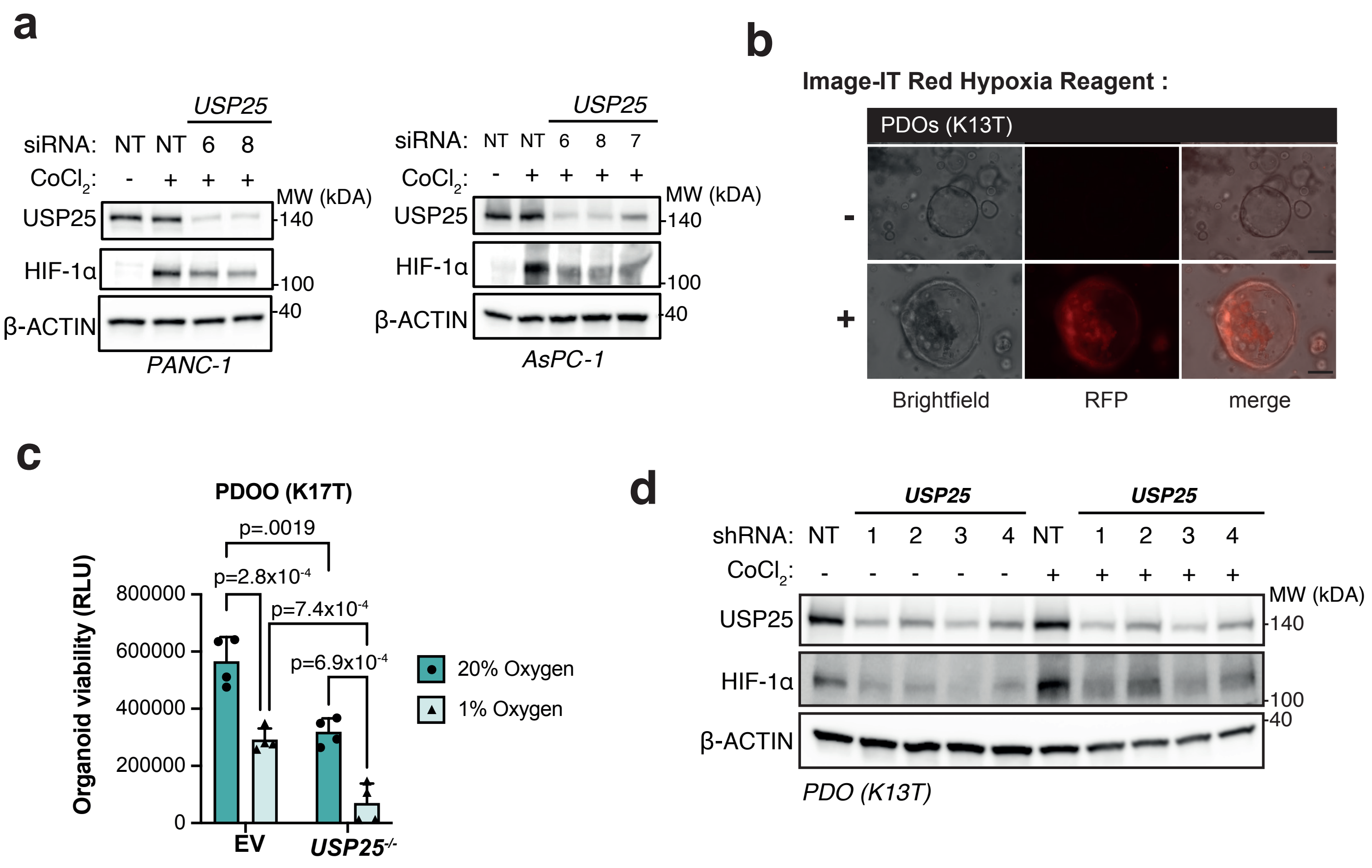

# Supplementary Figure 8

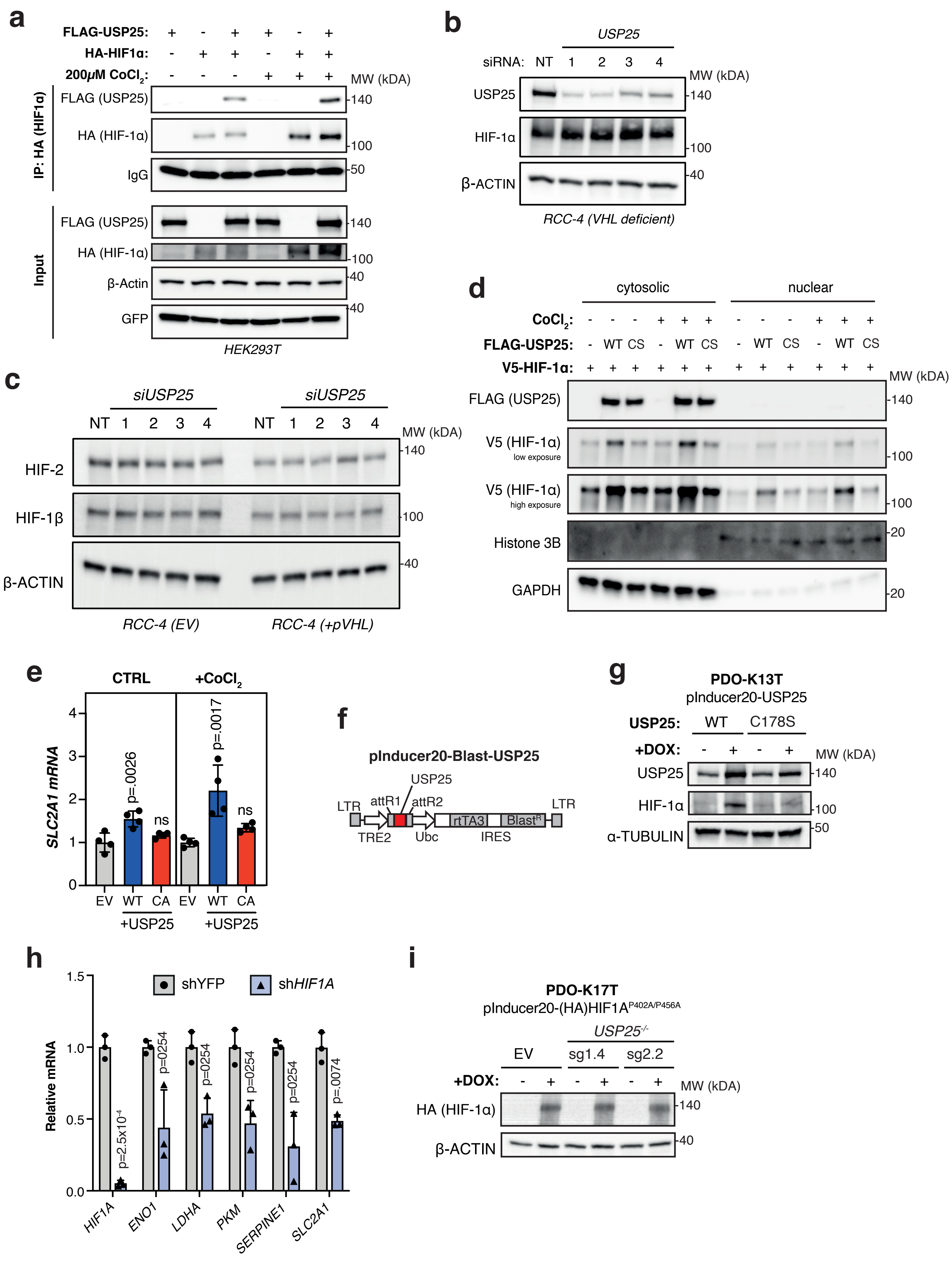

# Supplementary Figure 9

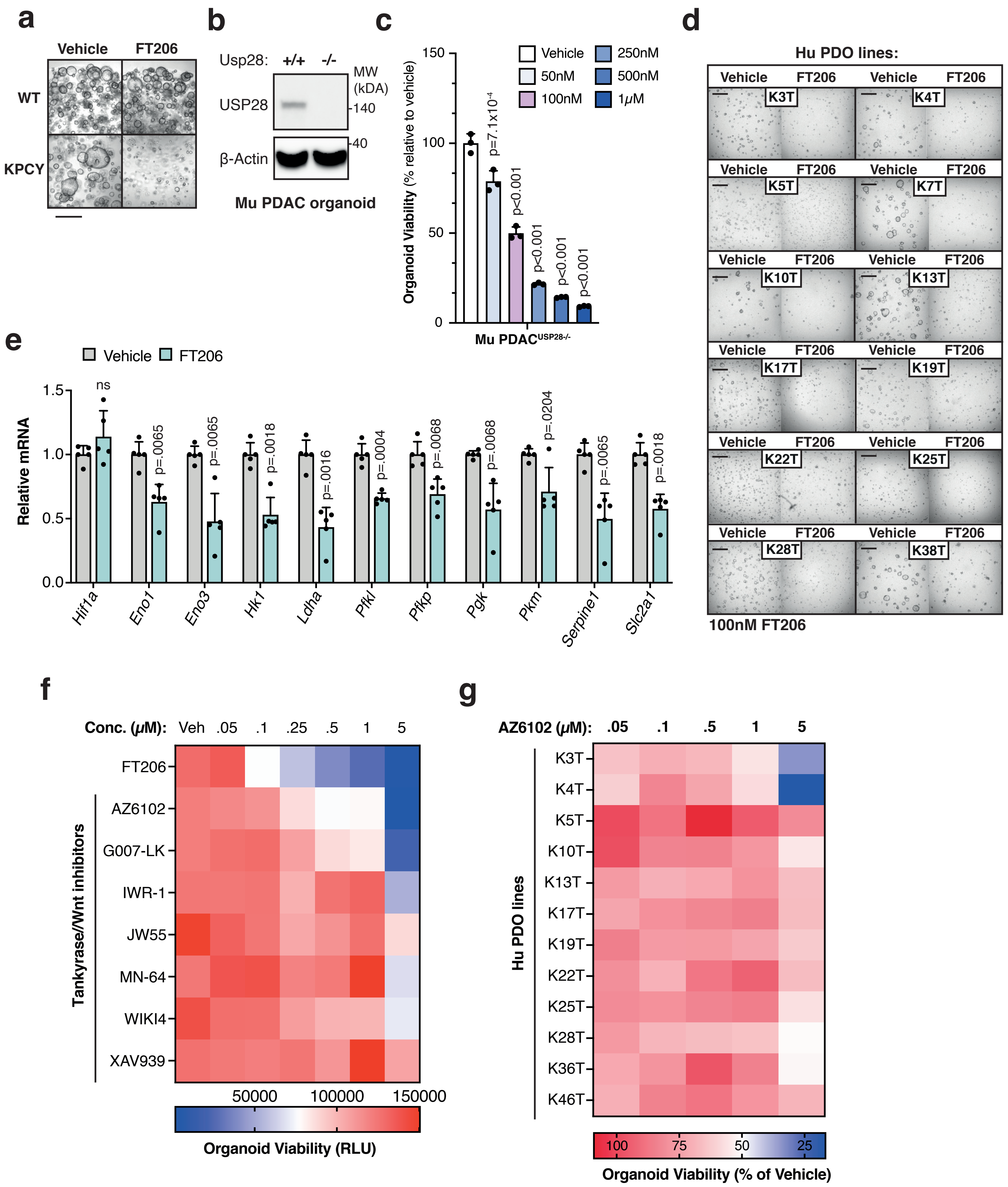

# Supplementary Figure 10

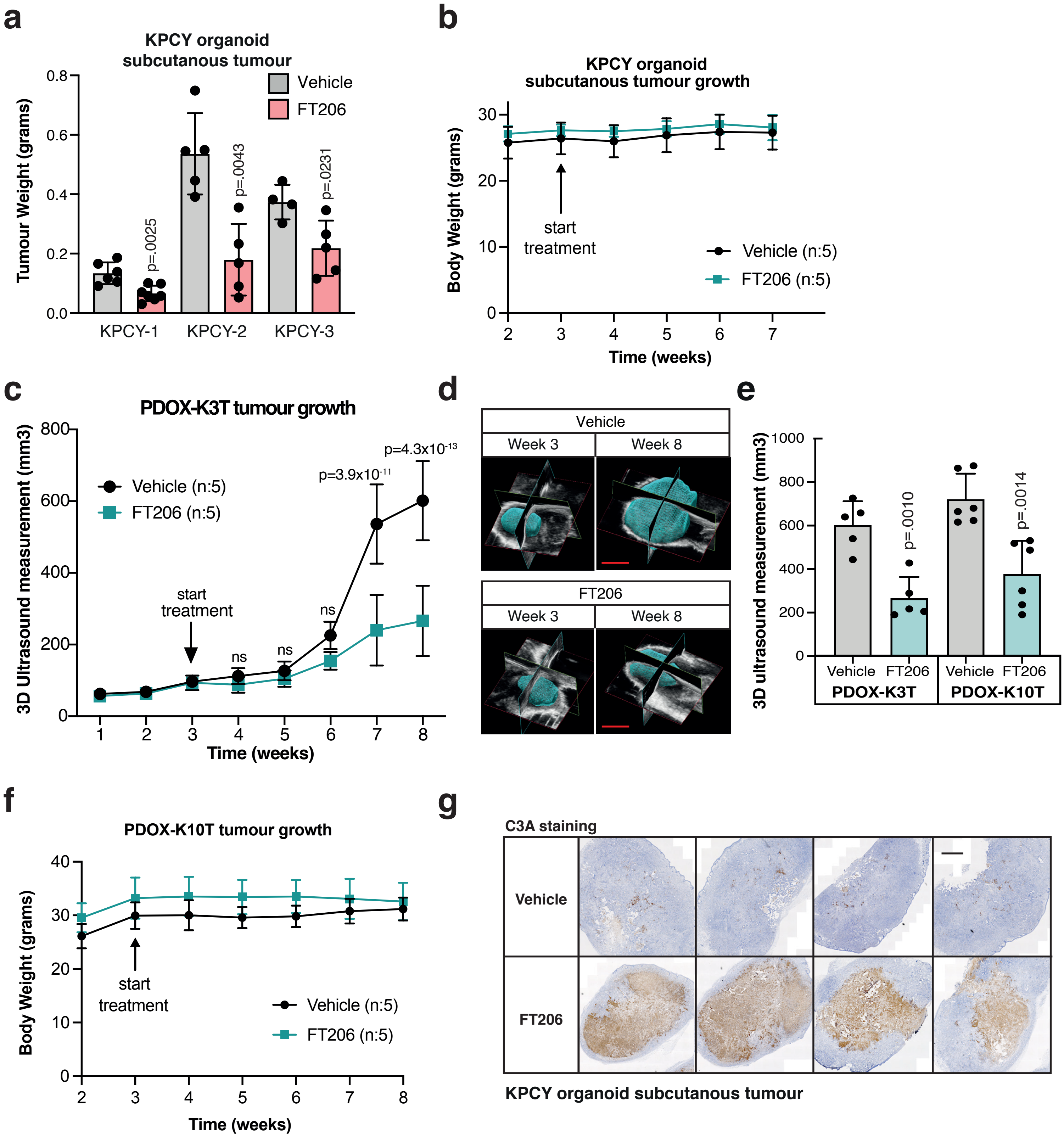

# Supplementary Figure 11

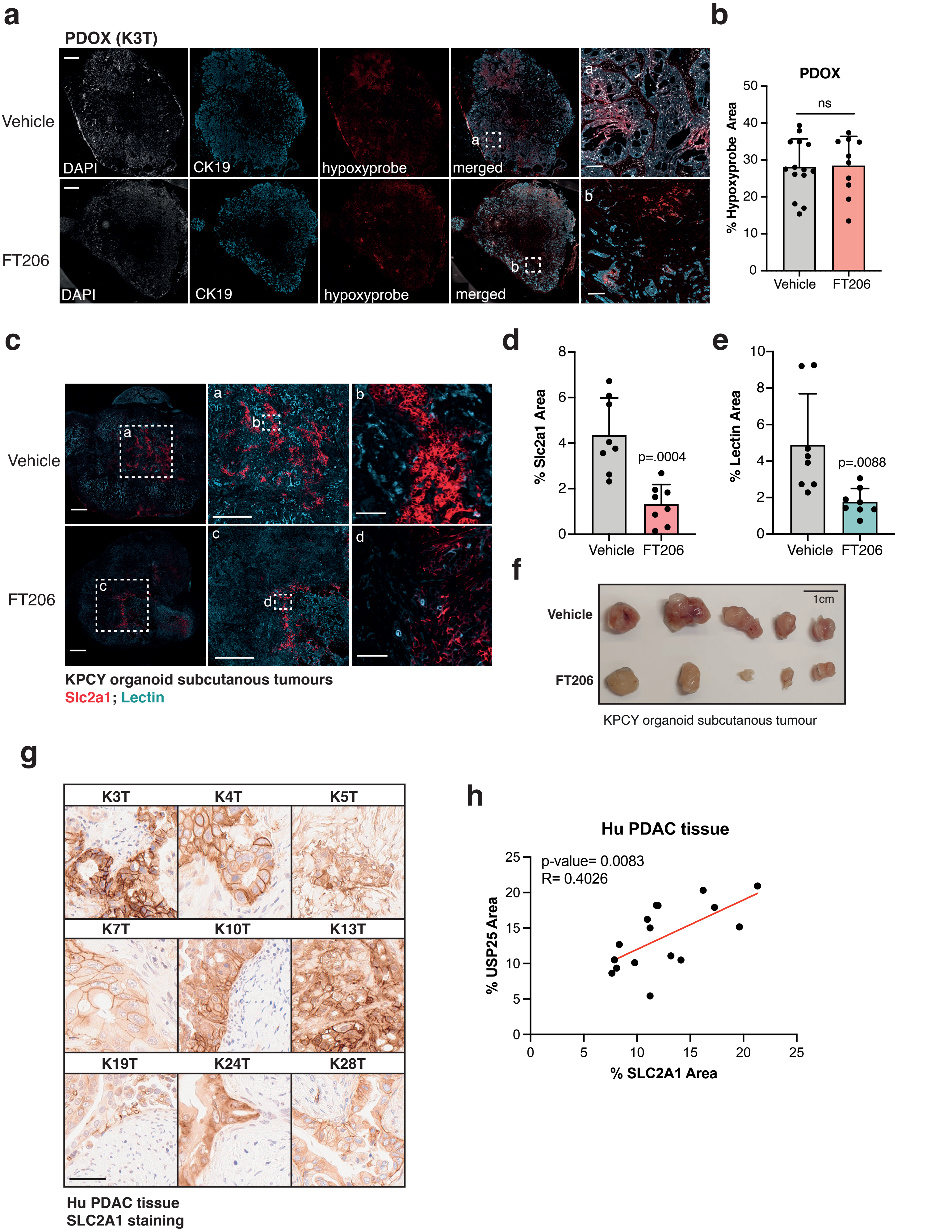

# Supplementary Figure 12

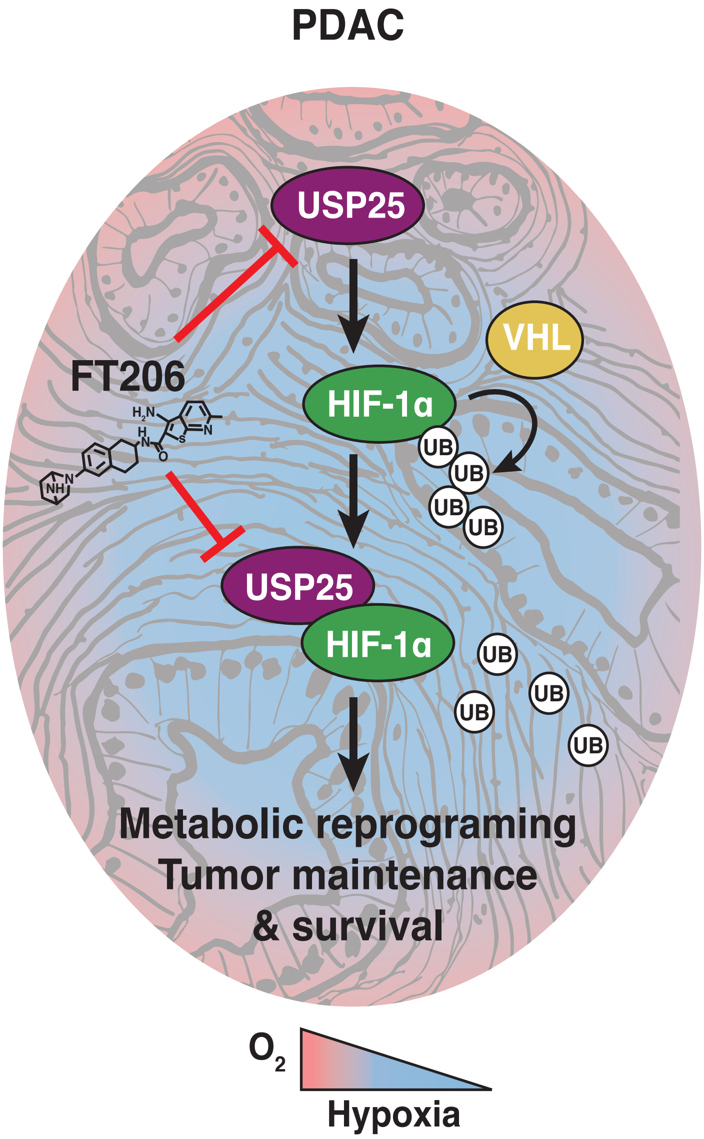

Supplement: Supplementary file 1 — Supplementary Information [file 41467_2022_29684_MOESM1_ESM.pdf]
