# Supplementary material for: USP25 promotes pathological HIF-1-driven metabolic reprogramming and is a potential therapeutic target in pancreatic cancer
Source: Nat Commun. 2022 Apr 19;13:2070. doi: 10.1038/s41467-022-29684-9 (PMC9018856; doi:10.1038/s41467-022-29684-9)
Supplement: Supplementary file 2 — Reporting Summary [file 41467_2022_29684_MOESM2_ESM.pdf]

nature portfolio

Corresponding author(s): Axel Behrens
Last updated by author(s): Feb 28, 2022

Reporting Summary

Nature Portfolio wishes to improve the reproducibility of the work that we publish. This form provides structure for consistency and transparency in reporting. For further information on Nature Portfolio policies, see our Editorial Policies and the Editorial Policy Checklist.

Statistics

For all statistical analyses, confirm that the following items are present in the figure legend, table legend, main text, or Methods section.

n/a	Confirmed	
☐	☒	The exact sample size (n) for each experimental group/condition, given as a discrete number and unit of measurement
☐	☒	A statement on whether measurements were taken from distinct samples or whether the same sample was measured repeatedly
☐	☒	The statistical test(s) used AND whether they are one- or two-sided<br>*Only common tests should be described solely by name; describe more complex techniques in the Methods section.*
☐	☒	A description of all covariates tested
☐	☒	A description of any assumptions or corrections, such as tests of normality and adjustment for multiple comparisons
☐	☒	A full description of the statistical parameters including central tendency (e.g. means) or other basic estimates (e.g. regression coefficient) AND variation (e.g. standard deviation) or associated estimates of uncertainty (e.g. confidence intervals)
☐	☒	For null hypothesis testing, the test statistic (e.g. F, t, r) with confidence intervals, effect sizes, degrees of freedom and P value noted<br>*Give P values as exact values whenever suitable.*
☒	☐	For Bayesian analysis, information on the choice of priors and Markov chain Monte Carlo settings
☒	☐	For hierarchical and complex designs, identification of the appropriate level for tests and full reporting of outcomes
☐	☒	Estimates of effect sizes (e.g. Cohen's d, Pearson's r), indicating how they were calculated

Our web collection on statistics for biologists contains articles on many of the points above.

Software and code

Policy information about availability of computer code

Data collection	High-content phenotypic monitoring: lncucyte S3 system (Sartorius); 3D ultrasound imaging: Microscan transducer MS-550D & 3D stage onrorl system (VEVO 2100; Visualsonics); quantitative PCR: 7500 fast and Via? (Applied Biosystems); RNA sequencing: lllumina HiSeq 4000 (lllumina); immunoblot: Fuji LAS4000 (GE Healthcare); Mass spectrometry: Orbitrap Fusion Lumos Tribrid (ThermoFisher); immunohistochemistry and fluorescent images: Zeiss Axio Scan.Zl; RNA-seq: RNA-seq: lllumina HiSeq 4000; metabolite extraction & gas chromoatography-mass spectrometry: Agilent 7890B-7000C GC-MSD (Agilent)
Data analysis	Graph Pad Prism 9; lmageJ 1.5; Zen 3.0; Perseus v2.0.3.0 (Tyanova et al., 2016); MaxQuant v2.0.3.1 (Cox et al., 2016); Trimmomatic 0.36 (Bolger et al., 2014); RSEM package v.1.2.31 (Li and Dewey, 2011); STAR alignment algorithm v.2.5.2a (Dobin and Gingeras, 2015); DESeq2 package v.1.20.0 (Love et al., 2014); pcaMethods package v.1.82.0 (Thevenot et al., 2015); MANIC software version 1.0 (Behrends et al., 2011); Km plotter (Nagy et al., 2018); GEPIA (Tang et al., 2017); Cluster 3.0 (De Hoon et al., 2004); Metascape (Zhou et al., 2019); Vevo lab software version 3.2.0.

For manuscripts utilizing custom algorithms or software that are central to the research but not yet described in published literature, software must be made available to editors and reviewers. We strongly encourage code deposition in a community repository (e.g. GitHub). See the Nature Portfolio guidelines for submitting code & software for further information.

Data

Policy information about availability of data

All manuscripts must include a data availability statement. This statement should provide the following information, where applicable:

- Accession codes, unique identifiers, or web links for publicly available datasets
- A description of any restrictions on data availability
- For clinical datasets or third party data, please ensure that the statement adheres to our policy

The RNA sequencing dataset is available on the GEO database, GSE166077. The mass spectrometry proteomics data have been deposited to the ProteomeXchange Consortium via the PRIDE partner repository with the dataset identifier PXD023424. All other data is available in the Source Data File.

Field-specific reporting

Please select the one below that is the best fit for your research. If you are not sure, read the appropriate sections before making your selection.

☒ Life sciences ☐ Behavioural & social sciences ☐ Ecological, evolutionary & environmental sciences

For a reference copy of the document with all sections, see nature.com/documents/nr-reporting-summary-flat.pdf

Life sciences study design

All studies must disclose on these points even when the disclosure is negative.

Sample size	No statistical methods were used to predetermine sample size. Sample size was determined to be adequate based on the magnitude and consistency of measurable differences between groups. In the case of animal experiments we struck a balance between reducing the number of animals and attaining statistically significant results.
Data exclusions	No data were excluded from analysis.
Replication	All experiments were repeated multiple times and/or on multiple biological replicates with similar results as indicated in the figure legends.
Randomization	For the in vivo subcutaneous tumor models, tumor bearing mice were randomized based on the starting tumor size. For all other experiments randomization was not performed because the analyses was done according to genotype or treatment.
Blinding	Where possible, samples were labeled with a code rather than with the genotype or treatment.

Reporting for specific materials, systems and methods

We require information from authors about some types of materials, experimental systems and methods used in many studies. Here, indicate whether each material, system or method listed is relevant to your study. If you are not sure if a list item applies to your research, read the appropriate section before selecting a response.

Materials & experimental systems

n/a	Involved in the study	
☐	☒	Antibodies
☐	☒	Eukaryotic cell lines
☒	☐	Palaeontology and archaeology
☐	☒	Animals and other organisms
☐	☒	Human research participants
☒	☐	Clinical data
☒	☐	Dual use research of concern

Methods

n/a	Involved in the study	
☒	☐	ChIP-seq
☒	☐	Flow cytometry
☒	☐	MRI-based neuroimaging

Antibodies

Antibodies used	Polyclonal rabbit anti-USP25 (Sigma-Aldrich, HPA024142); Polyclonal rabbit anti-USP25 (Abcam, Ab187156); Polyclonal rabbit anti-SLC2A1/Glut1 (Alpha Diagnostics, GT11-A); Polyclonal rabbit anti human/mouse active Caspase-3 (R&D Systems, AF835); Monoclonal mouse anti-aSMA (Sigma-Aldrich, A5228); Monoclonal rat anti-CK19 (Developmental Studies Hybridoma Bank, TROMA-III); Polyclonal goat anti-GFP (recognizes YFP) (Abcam, ab6673); Monoclonal mouse anti-b-actin (AC-15), directly conjugated to HRP (Abcam, Ab49900); Polyclonal rabbit anti-GAPDH (Abcam, Ab9485); Polyclonal rabbit anti-Histone 3B (Millipore, 06755); Polyclonal rabbit anti-HIF1a (Novus, NB100-449); Polyclonal rabbit anti-HIF-2 (Cell Signaling, 7096); Polyclonal rabbit anti-ARNT (Cell Signaling, 5537); Polyclonal rabbit anti-c-MYC (Abcam, Y69 clone, ab32072); Polyclonal rabbit anti-USP28 (Sigma, HP006779); Monoclonal mouse anti-FLAG (M2), conjugated to HRP (Sigma-Aldrich, A8592); Polyclonal anti-HA (Sigma-Aldrich, H6908); Polyclonal anti-VHL (Cell Signaling, 68547); Monoclonal mouse anti-V5 (tag) (Invitrogen, R960-25); Lycopersicon Esculentum (Tomato) Lectin-DyLight-488

(Vector Laboratories, DL-1174); Donkey anti rat IgG Alexa Fluor488 (Life Technology, A21208); Donkey anti mouse IgG Alexa Fluor546 (Life Technology, A10036); Donkey anti rabbit IgG Alexa Fluor647 (Life Technology, A31573); monoclonal rabbit IgG, Isotype control (Abeam, Ab172730); EZview red anti-HA affinity gel (Sigma-Aldrich, E6779); Dynabeads (M-280) sheep anti-rabbit IgG (Invitrogen, 11203D); High Capacity Neutravidin Agarose resin (Thermo Fisher Scientific,29204).

Validation

All antibodies were commercially validated (see manufacturer's website reported below), previously published or validated in the current study (see below).

Polyclonal rabbit anti-USP25 (Sigma-Aldrich, HPA024142); https://www.sigmaaldrich.com/GB/en/product/sigma/hpa024142. Validated in tissues for IHC (Figure 3a) and also online with shRNA-mediated knockdown in cells.
Polyclonal rabbit anti-USP25 (Abcam, Ab187156); https://www.abcam.com/usp25-antibody-epr15019-ab187156.html. Validated for WB with shRNA-mediated knockdown in cells (Figure 4c) and CRISPR-cas9 knockout in cells (Figure 4j).
Polyclonal rabbit anti-SLC2A1/Glut1 (Alpha Diagnostics, GT11-A); https://www.4adi.com/4adi/rabbit-anti-mouse-glucose-transp-1-glut-1-igg-1-aff-pure-11522-p.html. Validated for IHC in Sup Figure 11g, and for IF in Figure 7j and Sup Figure 11c.
Polyclonal rabbit anti human/mouse active Caspase-3 (R&D Systems, AF835); https://www.rndsystems.com/products/human-mouse-active-caspase-3-antibody_af835. See validation images online and Figure 7g.
Monoclonal mouse anti-aSMA (Sigma-Aldrich, A5228); https://www.sigmaaldrich.com/GB/en/product/sigma/a5228. See validation images online, and Figure 7j.
Monoclonal rat anti-CK19 (Developmental Studies Hybridoma Bank, TROMA-III); https://dshb.biology.uiowa.edu/TROMA-III. See validation images online, and Figure 3b, 7j, Supp Figure 11a.
Polyclonal goat anti-GFP (recognizes YFP) (Abcam, ab6673); https://www.abcam.com/GFP-antibody-ab6673.html?gclsrc=aw.ds|aw.ds&gclid=CjwKCAiAgvKQBhBbEiwAaPQw3EXaoZOnkclUWfimCMzcp0TZj127Hmo0U9sQ7zIZGkQXrvhDAGYF0BoCSuMQAvD_BwE. See validation images online, and Figure 3b for IHC and Supp Figure 8a for WB.
Monoclonal mouse anti-b-actin (AC-15), directly conjugated to HRP (Abcam, Ab49900); https://www.abcam.com/hrp-beta-actin-antibody-ac-15-ab49900.html. See validation online, and Figure 4 and 6.
Polyclonal rabbit anti-GAPDH (Abcam, Ab9485); https://www.abcam.com/gapdh-antibody-loading-control-ab9485.html. See validation images online, and Supp Figure 8d.
Polyclonal rabbit anti-Histone 3B (Millipore, 06755); https://www.merckmillipore.com/GB/en/product/Anti-Histone-H3-Antibody,MM_NF-06-755. See validation images online, and Supp Figure 8d.
Polyclonal rabbit anti-HIF1a (Novus, NB100-449); https://www.novusbio.com/products/hif-1-alpha-antibody_nb100-449. WB validation online, and Figure 6b, 6f-k, Supp Figure 7a, 7d, 8b.
Polyclonal rabbit anti-HIF-2 (Cell Signaling, 7096); https://www.cellsignal.co.uk/products/primary-antibodies/hif-2a-d9e3-rabbit-mab/7096. WB validation online, and Figure 6b, Supp Figure 8c.
Polyclonal rabbit anti-ARNT (Cell Signaling, 5537); https://www.cellsignal.co.uk/products/primary-antibodies/hif-1b-arnt-d28f3-xp-rabbit-mab/5537. WB validation online, and Figure 6b, 6f-k, Supp Figure 8b.
Polyclonal rabbit anti-c-MYC (Abcam, Y69 clone, ab32072); https://www.abcam.com/c-myc-antibody-y69-ab32072.html. WB validation online, and Figure 6b.
Polyclonal rabbit anti-USP28 (Sigma, HP006779); https://www.sigmaaldrich.com/GB/en/product/sigma/hpa006779. WB validation online, and Supp Figure 9b.
Monoclonal mouse anti-FLAG (M2), conjugated to HRP (Sigma-Aldrich, A8592); https://www.sigmaaldrich.com/GB/en/product/sigma/a8592. Validated online, and Supp Figure 8a, 8d.
Polyclonal anti-HA (Sigma-Aldrich, H6908); https://www.sigmaaldrich.com/GB/en/product/sigma/h6908. WB validation online, and Supp Figure 8a.
Polyclonal anti-VHL (Cell Signaling, 68547); https://www.cellsignal.co.uk/products/primary-antibodies/vhl-antibody/68547. Validation for WB is online, and Figure 6g.
Monoclonal mouse anti-V5 (tag) (Invitrogen, R960-25); https://www.thermofisher.com/antibody/product/V5-Tag-Antibody-Monoclonal/R960-25. Validation for WB online, and Figure Supp Figure 8d.

Eukaryotic cell lines

Policy information about cell lines

Cell line source(s)	Cell Services of the Francis Crick Institute for HEK293T, RCC-4, PANC-1, and AsPC-1
Authentication	All cells lines were authenticated using STR profiling and results checked against any available STR data for the parent line using commercial banks.
Mycoplasma contamination	All cell lines were obtained mycoplasma free from Cell Services of the Francis Crick Institute
Commonly misidentified lines (See ICLAC register)	No commonly misidentified cell lines were used in this study.

Animals and other organisms

Policy information about studies involving animals; ARRIVE guidelines recommended for reporting animal research

Laboratory animals	The following mouse lines were used in this study: KrasLSL-G12D (Jackson, 2001), Trp53flox (Marino, 2000), Pdxl-Cre (Hingorani, 2003), Pdxl-Flp, KRasFSF-G12D, Trp53frt, Rosa26FSF-CreER (Schiinhuber, 2014), Rosa26mT/mG (Muzumda, 2007) and Usp28flox (Diefenbacher, 2014) Rosa26LSL-YFP (Srinivas, 2001). These lines were inter-crossed to generate the desired genotypes on a C57BL/6 background. 5-8 weeks old Pdxl-Cre;KrasLSL-G12D;Trp53fl/fl;Rosa26LSL-YFP, 8-10 weeks old Pdxl-Flp;KrasFSF-G12D;Trp53frt/frt;Rosa26FSF-CreER;Rosa26mT/mG;GUsp28flox/flox, and 7-12 weeks old male NSG and Nu/Nu mice were used for the experiments.
Wild animals	The study did not involve wild animals.

Field-collected samples

The study did not involve samples collected from the field.

Ethics oversight

All animal experiments were approved by the Francis Crick Institute Animal Ethics Committee and conformed to UK Home Office regulations under the Animals (Scientific Procedures) Act 1986 including Amendment Regulations 2012.

Note that full information on the approval of the study protocol must also be provided in the manuscript.

Human research participants

Policy information about studies involving human research participants

Population characteristics

Patient Line/TNM Stage/Histology/ Neoadjuvant Chemo:
K3T/T2N1M0/ Poorly differentiated/None
K4T/T2N2M0/ Moderately differentiated/None
K5T/T3N2M0/Moderately differentiated/ None
K7T/T4N1M0/Moderately differentiated/FOLFIRINOX 12 Cycles
KIOT/T2N1M0/ Moderately differentiated/None
K13T/T2N1M0/ Moderately differentiated/ None
K17T/ Unknown/ Moderately differentiated/ None
K19T/Unknown/ Moderately differentiated/ None
K22T/T2N1M0/ Moderately differentiated/ None
K25T/T2N1M0/ Moderately differentiated/ None
K28T/T2N1M0/ No diff. status/None
K38T/T2N1M0/ No diff. status/ None
K46T/T2N2M0/ Moderately differentiated/ None

Recruitment

Patients were recruited by the research nursing staff at King's College Hospital in London, United Kingdom. Participants were recruited by screening pre-assessment clinic lists or multidisciplinary team lists to identify patients who were undergoing pancreatic surgery for radiologically diagnosed primary pancreatic cancers with features most consistent with pancreatic adenocarcinoma. Patients were identified prospectively based on these criteria and consented prior to their day of surgery. Tissue was then obtained from these patients who underwent surgical resections. The sizable majority (>80%) of patients approached were consented, suggesting a limited impact of self-selection, given consent did not involve anything clinically beyond usual routine care.

The main bias in our study was a selection bias for surgical candidates only (Stage I/II disease). We did not have ethical approval to obtain tissue from non-surgical candidates undergoing biopsy of metastatic or locally advanced tumors (stage III/IV disease). As a result, our population only represents a subset of pancreatic cancer patients. It is possible that USP25 dependence may not be as significant a factor in human stage III/IV patients. However, we emphasis nonetheless that USP25 dependence as exhibited in advanced murine genetic models and across a wide array of PDO models with distinct in vitro and genetic phenotypes.

Ethics oversight

The study was carried out with formal ethical approval from the Research Ethics Committee (REC, Northern Ireland office) and Health Research Authority (HRA). The study reference ID is IRAS #199628, 16/NI/0119. The study is compliant with all relevant ethical regulations regarding research involving human participants.

Note that full information on the approval of the study protocol must also be provided in the manuscript.
